# QQ-SNV: single nucleotide variant detection at low frequency by comparing the quality quantiles

**DOI:** 10.1186/s12859-015-0812-9

**Published:** 2015-11-10

**Authors:** Koen Van der Borght, Kim Thys, Yves Wetzels, Lieven Clement, Bie Verbist, Joke Reumers, Herman van Vlijmen, Jeroen Aerssens

**Affiliations:** 1 Janssen Infectious Diseases-Diagnostics BVBA, B-2340 Beerse, Belgium; 2 Interuniversity Institute for Biostatistics and statistical Bioinformatics, Katholieke Universiteit Leuven, B-3000 Leuven, Belgium; 3 Ghent University, Applied Mathematics, Informatics and Statistics, B-9000 Ghent, Belgium

**Keywords:** Classifier model, Illumina deep sequencing, Logistic regression, Single nucleotide variant, *True* variant

## Abstract

**Background:**

Next generation sequencing enables studying heterogeneous populations of viral infections. When the sequencing is done at high coverage depth (“deep sequencing”), low frequency variants can be detected. Here we present QQ-SNV (http://sourceforge.net/projects/qqsnv), a logistic regression classifier model developed for the Illumina sequencing platforms that uses the quantiles of the quality scores, to distinguish *true* single nucleotide variants from sequencing errors based on the estimated SNV probability. To train the model, we created a dataset of an *in silico* mixture of five HIV-1 plasmids. Testing of our method in comparison to the existing methods LoFreq, ShoRAH, and V-Phaser 2 was performed on two HIV and four HCV plasmid mixture datasets and one influenza H1N1 clinical dataset.

**Results:**

For default application of QQ-SNV, variants were called using a SNV probability cutoff of 0.5 (QQ-SNV_D_). To improve the sensitivity we used a SNV probability cutoff of 0.0001 (QQ-SNV_HS_). To also increase specificity, SNVs called were overruled when their frequency was below the 80^th^ percentile calculated on the distribution of error frequencies (QQ-SNV_HS-P80_).

When comparing QQ-SNV versus the other methods on the plasmid mixture test sets, QQ-SNV_D_ performed similarly to the existing approaches. QQ-SNV_HS_ was more sensitive on all test sets but with more false positives. QQ-SNV_HS-P80_ was found to be the most accurate method over all test sets by balancing sensitivity and specificity. When applied to a paired-end HCV sequencing study, with lowest spiked-in *true* frequency of 0.5 %, QQ-SNV_HS-P80_ revealed a sensitivity of 100 % (*vs.* 40–60 % for the existing methods) and a specificity of 100 % (*vs.* 98.0–99.7 % for the existing methods). In addition, QQ-SNV required the least overall computation time to process the test sets.

Finally, when testing on a clinical sample, four putative *true* variants with frequency below 0.5 % were consistently detected by QQ-SNV_HS-P80_ from different generations of Illumina sequencers.

**Conclusions:**

We developed and successfully evaluated a novel method, called QQ-SNV, for highly efficient single nucleotide variant calling on Illumina deep sequencing virology data.

**Electronic supplementary material:**

The online version of this article (doi:10.1186/s12859-015-0812-9) contains supplementary material, which is available to authorized users.

## Background

Next generation sequencing (NGS) at high coverage depth (deep sequencing) has proven to be valuable within clinical virology [1–4]. In order to guide the treatment of HIV-1 patients, the viral DNA is routinely sequenced to study the accumulation over time of resistance associated mutations to antiretroviral drugs. With the advent of NGS technologies, single nucleotide variants (SNVs) present at low prevalence (minor variants) can be detected, whereas with traditional Sanger sequencing, SNVs could be identified only when present in >20 % of the genomes in a patient’s sample. However, as NGS technologies are error-prone, *true* variants need to be distinguished from sequencing errors, which is especially challenging for low frequency variations.

Currently, the Illumina sequencing systems are dominating the NGS market [5] and novel methodologies to accurately and efficiently determine genotypic variations from ultra-deep sequencing (UDS) data for this technology of sequencers are needed. Moreover, the NGS system that has in the past most often been used within virology, i.e. the Roche 454 pyrosequencing [6] system, will be phased-out by 2016.

Over the last several years multiple groups have devised algorithms to perform SNV calling on UDS data: ShoRAH [7], V-Phaser 2 [8], LoFreq [9], deepSNV [10], and RVD [11, 12]. Whereas the latter two rare variant detection approaches can robustly detect *true* variants with a prevalence of 0.1 %, the use of these so-called ultrasensitive methods is limited to experiments where reference control samples have been included. The former two algorithms represent 454 legacy methods. Given that for the Illumina sequencing systems most of the errors observed are mismatches, rather than insertion or deletion errors [13] which are the most prominent errors in pyrosequencing-based NGS data, these methods may not work optimal for Illumina NGS data. Lastly, the LoFreq method constitutes an approach that is developed for variant analysis that calculates p-values assuming a Poisson binomial distribution as null model for the number of variant bases at a position in the alignment. In this method, the Illumina sequencer quality values associated to each base in a column are taken as input for the null model. While LoFreq is efficient in detecting low frequency SNVs in high-coverage datasets, efficiency decreases with both the coverage depth of the dataset and the variant percentage. This means that LoFreq will need more runtime e.g. to detect a variant at 10 % than at 0.5 % at the same coverage depth.

In this manuscript, we describe Quality Quantile-SNV (QQ-SNV), a novel logistic regression method for the highly sensitive and specific detection of *true* variants from Illumina UDS data without the need to incorporate reference control samples in the sequencing experiment. As opposed to LoFreq we do not model per base the variant frequency but apply a trained logistic regression model. Therefore, our novel method does not have the counterintuitive and undesirable property of being less efficient in detecting variants at higher frequency. Instead of considering all types of variations, such as indels (as e.g. in [8]), our aim was to develop a novel SNV caller for detection of *true* single nucleotide variants only (cf in [7] and [9]), that is applicable to NGS data of any read length from different Illumina sequencers. Different from most of the existing algorithms, QQ-SNV utilizes primarily the Illumina quality scores associated with individual nucleotides by the sequence basecaller. This approach consequently enables to effectively deal with the alignment of raw sequence data (“reads”) without any filtering or trimming applied. To improve the classification performance (“no error” *vs.* error) of QQ-SNV we first recalibrated the “raw” quality scores (Additional file 1: Figures S1 and S2) by means of a companion logistic regression “recalibration” model. After recalibration [14–16], less discrete (or more continuous) quality score values are obtained (Additional file 1: Figures S3 an S4) and the quality score distribution for the errors is more distinct from the quality score distributions obtained for the nucleotide reference or *true* variants as seen from the density plots and Quality Quantile plots. This led to the selection of more stable and information-rich quality score derived model parameters for the QQ-SNV classifier and convergence of the model fit. Moreover, as the sequencing error frequencies in forward and reverse reads differ [11, 17–19], the directionality of the reads is also taken into account within the regression.

## Data and methods

### Data

#### Sequencing

For the HIV and HCV plasmids datasets processed in-house for training and testing respectively, the Illumina Genome Analyzer IIx (GAIIx) was used for the paired-end sequencing. Basecalling was done using Bustard (CASAVA 1.7.0), the Illumina default basecaller, producing after de-multiplexing two FASTQ [20] files per plasmid: FASTQ/1 (reads 1 of paired-end reads) and FASTQ/2 (reads 2 of paired-end reads, sequenced after paired-end turn), containing the nucleotide sequences with a quality score assigned to each nucleotide. Training of the classifier model was then done on an *in silico* plasmid mixture dataset which we created with known *true* variant percentages.

#### Training data

##### FASTQ/1 Illumina GAIIx retrieved HIV plasmid sequence data

For the training of the classifier model the HIV PR-RT region (nt 1–1497) was sequenced. Five HIV-1 plasmids, with different mutational and error profile in the HIV protease-reverse transcriptase region (PR-RT) were processed together in one lane. The length of the reads was 51 base pairs. As the quality of the FASTQ/2 reads is typically lower than for the FASTQ/1 reads, for training of the QQ-SNV classifier model and the companion quality score recalibration model we used the FASTQ/1 data only. This allowed for better estimation of the model parameters with applicability of the model on paired-end or single-end data. Whereas the recalibration model was directly trained on the FASTQ/1 data, to train the classifier model the FASTQ/1 data was first mixed *in silico* (see next section).

The mapping of the FASTQ/1 reads was done per plasmid, where as reference sequence a HXB2 wild type background was used with the respective plasmid inserted in the region, at nucleotide position 2253–3749, where the *true* sequence was known. Within this 1497 bp region, 12 SNV positions contained a *true* variant (Additional file 2: Table S1), hence all differences to the HXB2 reference seen at other positions are *true* errors. Note that for this read mapping, we opted to not make a choice between the different read mapping tools [21]. Instead, regular expressions (perl) were used to search for the best possible ungapped alignment for the reads in forward or reverse direction. In total 1,183,162 reads covering the region 2253-3749 and having < 20 % mismatch nucleotide errors (including mismatch errors caused by not considering insertions or deletions) were retained for training of the quality score recalibration model. In this training data an equal percentage of reads in forward and reverse direction was observed (Additional file 2: Table S2). Coverage of the reads was comparable for all plasmids, with a similar average coverage range: 8388–9871 reads with exception of plasmid 2 where the average coverage was found to be lower (N = 3788 reads) (Additional file 1: Figure S5).

In Additional file 2: Table S3, for each of the five plasmids, the total number of errors observed in the region 2253–3749 is given as well as the per plasmid PHRED quality scores calculated from the plasmid error probabilities, where a high quality score indicates a smaller probability of error.

##### In silico HIV plasmid mixtures data set for training the QQ-SNV classifier

To train a model with high sensitivity and specificity to detect *true* variants present at low frequency, a dataset (hereafter called the ground set) was generated of 960 HIV plasmid mixture re-samplings of the FASTQ/1 reads from five plasmids (described in the previous section), as mapped in the region 2253–3749 (Additional file 1: Figure S5, Additional file 2: Table S2). For each re-sampling, reads were mixed *in silico* from a multinomial distribution in the plasmid mixture proportion 63, 30, 5, 1.5, and 0.5 %, and having a coverage depth of N = 5000. In total, the number of *true* variants in the ground set was 11,520 (=960 × 12), with *true* variant percentages populated in the range [0.22–100 %] (Additional file 1: Figure S6). In order to obtain a larger coverage depth for training of the QQ-SNV model ten re-samplings in the ground set were repeated for the same plasmid mixture proportion and their read mappings were combined. This resulted in a training dataset consisting of 96 read sets (training samples) with a coverage depth of 50,000 reads at each nucleotide position.

#### Test data

##### HIV plasmid mixture dataset 1

The HIV PR-RT region (nt 1–3332) was sequenced using the Illumina GAIIx sequencer with a single-end library layout mixing 6 plasmids at the ratio of 88.889, 10, 1, 0.1, 0.01, and 0.001 % [10]. The FASTQ file was obtained from the European Nucleotide Archive (ERX097243), and the length of the reads was 31 base pairs. Mapping to the HXB2 reference sequence (nt 2044–3615) was done using CLC Genomics Workbench (CLC bio, Aarhus, Denmark) using the default alignment settings. The average coverage depth of the plasmid mixture in the PR-RT region considered for the analysis (nucleotide position 2253–3584) was 103,142 reads.

##### HIV plasmid mixture dataset 2

The HIV IN region (nt 145–545) was sequenced using the Illumina HiSeq 2000 sequencer with a single-end library layout mixing 6 plasmids at the ratio of 60, 33.4, 5, 1, 0.5, and 0.1 % [22]. The FASTQ file was obtained from the European Nucleotide Archive (PRJEB5053), and the length of the reads was 50 base pairs. Mapping to the HXB2 reference sequence (nt 4349–4799) was done using CLC Genomics Workbench (CLC bio, Aarhus, Denmark) using the default alignment settings. The average coverage depth of the plasmid mixture in the IN region considered for the analysis (nucleotide position 4374–4774) was 3,857,069 reads.

##### HCV plasmid mixture datasets

The HCV NS3 region (nt 1–543) was sequenced using the Illumina GAIIx sequencer with a paired-end library layout. In the lab experiment, a HCV reference plasmid was mixed four times with a non-reference plasmid containing five *true* variants in the NS3 region (nucleotide position 3420–3962) considered for the analysis: 3525G > A, 3527C > G, 3882C > A, 3883G > A, and 3884G > A, where the spike-in percentage of the non-reference plasmid (and thus all five *true* variants) was 0.5, 1, 2, and 10 %, respectively. The four resulting HCV “spiked-in” plasmids were sequenced in different lanes, and the length of the reads was 70 base pairs. Mapping to the HCV subtype b reference sequence (nt 3420–3962, Genbank accession number AJ238799) was done using CLC Genomics Workbench (CLC bio, Aarhus, Denmark) using single-end alignment with the default settings, and the average coverage depth (reads 1 and 2) for the plasmids was 93,130 reads. Illumina sequencing data has been deposited in the European Nucleotide Archive under study accession number PRJEB5028. To test our method in comparison to the existing methods at different sequencing depths we generated lower coverage data by randomly downsampling the sequence alignment files (SAM files) of the four HCV “spiked-in” plasmid mixtures containing all paired-end reads using Picard command line tools v1.86 DownsampleSam. Reads were retained with a probability of 40, 10, 2.5 and 0.625 % resulting in average coverage depths of 37,270, 9291, 2295, and 561 reads, respectively.

##### H1N1 clinical dataset

The H1N1 neuraminidase (NA) reference and single-end alignments of the paired-end reads of the H1N1 BN3 clinical data sample, sequenced with both Illumina GAIIx (on one lane) and Illumina MiSeq, were obtained from [12]. The length of the reads was 37 base pairs. To test the performance in variant detection of QQ-SNV, the region at position 405–425 was considered, containing four SNVs called in [12], at the positions 410, 413, 415, and 421. After merging the alignments of the paired-end reads 1 and 2 using samtools v0.1.18 [23], the average coverage depth in this region was 360,359 reads and 36,812 reads for GAIIx and MiSeq, respectively.

No ethics approval was required for the use of the NGS data of the clinical isolate in this study. The BN3 clinical sample is one of the H1N1 infected patient samples of which the Illumina GAIIx and MiSeq sequencing data was made publicly available by Stanford University as publication material with the RVD method [12] on their server at URL: http://hamachi.stanford.edu/publication-material/rvd/clinical/.

### Methods

The goal of this paper is to build a logistic regression model to classify a nucleotide variant as SNV (*true* variant) or as error by exploiting the information that is in the distribution of the quality scores in the reads covering the variant location. Therefore in this section we first introduce the Illumina quality scores of which we assume that the empirical estimate of the distribution differs for *true* variants or nucleotide reference compared to an error at each location (Additional file 1: Figures S1 and S2). Second, we put forward our companion logistic regression “recalibration” model to recalibrate these quality scores with as purpose to further increase the “distance” between the quality score distribution of “no error” *vs.* error (Additional file 1: Figures S3 and S4). Third, we define the “distance metrics” which are derived from the per nucleotide recalibrated quality score distributions and are considered to be selected as predictors for the QQ-SNV classifier to model the probability of “no error” *vs.* error for each nucleotide at each position (Additional file 1: Figures S7-S9). Then, we describe how the *in silico* plasmid mixture QQ-SNV training data was generated by utilizing a resampling scheme and present the QQ-SNV model and how we selected and estimated the QQ-SNV model parameters by applying stepwise regression and weighted logistic regression, respectively. The latter approach corrects for imbalances in the training data (less SNVs than errors) and allows to use a probability threshold of 0.5 for default application of QQ-SNV. Next, we give the complete workflow for running the QQ-SNV classifier on new data, including the implementation of a high sensitivity threshold of 0.0001 and frequency-based variant filtering to increase sensitivity and reduce the number of false positives, respectively. Lastly, we give the parameter settings used for the competing SNV calling methods on the test datasets.

#### Quality scores and derived predictors

##### Quality scores

Illumina quality scores QUAL are retrieved from a FASTQ file and indicate the base call accuracy as assigned to the nucleotides by the basecaller software of the Illumina sequencer. Quality scores *Q* are so-called PHRED scores when they have the property of being logarithmically related to the base calling error probability *P*: *Q* = − 10 log_10_ *P*. While on the FASTQ/1 training data the llumina quality scores *QUAL* are in the *Q*-range 2–40, quality score recalibration (described in the next section) gave quality scores in the *Q*-range 1.62–27.6 – thus estimating the error probability *P* to be higher.

##### Recalibrated quality scores

While correct interpretation of the quality scores may not seem to be required when mainly used for constructing predictors in the QQ-SNV model, we found that quality score recalibration improved the QQ-SNV classification performance (Additional file 2: Table S4 and Additional file 1: Figure S10). When evaluating the QQ-SNV model generation procedure, a major observation was that more continuous quality score values led to the selection of more stable and information-rich quality score derived model parameters as well as convergence of the model fit and an increased “distance” between the quality score distribution of “no error” *vs.* error. Hence, we have integrated quality score recalibration in the QQ-SNV workflow by means of logistic regression, as previously employed in [14, 16]. Here, to calculate the recalibrated quality scores for deriving the predictors of the QQ-SNV model, we trained the following companion binary logistic regression model on the FASTQ/1 HIV plasmid sequence data (1,183,162 reads from five HIV plasmids, see section Training data) with as outcome Y_FASTQ/1_ = 0 (no error) or 1 (error):

$$ \log \kern0.3em  it\left(\mathrm{Probability}\ \mathrm{of}\ {Y}_{FASTQ/ 1}=1\right)={\alpha}_0+{\alpha}_1\times QUAL+{\alpha}_2\times RELPOS, $$

where *Y*_*FASTQ*/1 *i*,*j*,*k*,*r*_ =*I*{nucleotide *k* at position *j* in read *r* of sample *i* is a sequencing error} and the probability that *Y*_*FASTQ*/1 *i*,*j*,*k*,*r*_ is a sequencing error is calculated from the covariates *QUAL*_*i*,*j*,*k*,*r*_ and *RELPOS*_*i*,*j*,*k*,*r*_. *QUAL* is the original nucleotide Illumina PHRED quality score to be recalibrated. *RELPOS* is the distance of the nucleotide position in the read from the 3’ end, normalized by the read length. The recalibrated PHRED quality score is then calculated as: *Q. RECAL*_*i*,*j*,*k*,*r*_ = − 10 log_10_(Probability *Y*_*FASTQ*/1 *i*,*j*,*k*,*r*_ is a sequencing error).

The coefficients of the QQ-SNV recalibration model are given in Table 1 (confidence intervals in Additional file 3), and were fitted using a mixed effects model, using a random effect for factor PLASMID to take the difference in plasmid error probability into account (Additional file 2: Table S3) [24]. The random effect variance was 0.199.

**Table 1:** Quality score recalibration model trained on FASTQ/1 GAIIx data

Parameter	Coefficient	Significance (*P*-value)
Intercept	0.84 (*α*_0_)	2.27e-5
*QUAL*	−0.16 (*α*_1_)	<2e-16
*RELPOS*	−0.89 (*α*_2_)	<2e-16

##### Quality score derived predictors

Whereas the quality score recalibration model is a logistic regression model that predicts the probability of sequencing error at the nucleotide level for each individual read *r*, the QQ-SNV model calculates the probability of “no sequencing error” of a nucleotide *k* for all reads of a sample *i* mapped at a position *j*, using the recalibrated quality 100-quantiles (percentiles) *p* – calculated from multiple reads with the same direction *d* (forward/reverse) and containing the same nucleotide *k* (A/C/G/T) at an aligned position *j* – as summarized quality scores. The following “distance metrics” were derived per read direction from the per nucleotide recalibrated quality score distributions: *QQnorm. dir* calculates the recalibrated quality quantile percentages after normalization to the “per position *j*” recalibrated quality quantile (calculated irrespective of nucleotide) in the range [0,1] (Additional file 1: Figure S11), thus independent of the Illumina recalibrated Q-range (Additional file 1: Figure S4). *QQ. dir* then calculates per percentile *p* the nucleotide distance in the range [0,1] as the difference of the normalized nucleotide *k* recalibrated quality quantile from the reference value of 100 %, relative to the distance calculated for the nucleotide with the worst quality (Additional file 1: Figure S12). After that *D. dir* is calculated as the total distance summed over all nineteen percentiles *p* considered (Additional file 1: Figure S13). In the formula given below *QQnorm. dir*_*p*,*i*,*j*,*k*,*d*_, *QQ. dir*_*p*,*i*,*j*,*k*,*d*_, and *D. dir*_*i*,*j*,*k*,*d*_ are calculated for nucleotide *k* in the aligned reads for sample *i* at position *j* in a sequenced region of interest and per read direction *d*.

$$ \begin{array}{l} QQnorm.di{r}_{p,i,j,k,d}=\frac{percentil{e}_p\left(Q. RECA{L}_{i,j,k}\left| read. direction=d\right.\right)}{percentil{e}_p\left(Q. RECA{L}_{i,j}\left|\begin{array}{l} read. direction=d,\;\\ {} nt\in \left\{A,C,G,T,N\right\}\end{array}\right.\right)},\mathrm{with}\;p\in \left\{{5}^{th},\;{10}^{th}, \dots,\;{95}^{th}\right\},\;d\in \left\{ forward,\; reverse\right\}\hfill \\ {}QQ.di{r}_{p,i,j,k,d}=\frac{ \max \left(0,\;\left(1- QQnorm.di{r}_{p,i,j,k,d}\right)\right)}{\left(1-{ \min}_{nt=k}\left( QQnorm.di{r}_{p,i,j,k,d}\right)\right)}, with\;k\in \left\{A,C,G,T\right\}\hfill \\ {}D.di{r}_{i,j,k,d}={\displaystyle {\sum}_{p\in P}QQ.di{r}_{p,i,j,k,d}},\kern0.24em \mathrm{with}\;P=\left\{{5}^{th},\;{10}^{th}, \dots,\;{95}^{th}\right\},\;d\in \left\{ forward,\; reverse\right\}\hfill \end{array} $$

Next, the “distance metrics” selectable for the QQ-SNV model: *QQnorm* (Additional file 1: Figure S7), *QQ* (Additional file 1: Figure S8), and *D* (Additional file 1: Figure S9) are calculated as follows:

$$ \begin{array}{l} QQnor{m}_{p,i,j,k}= \min \left( QQnorm.di{r}_{p,i,j,k, forward}, QQnorm.di{r}_{p,i,j,k, reverse}\right)\hfill \\ {}Q{Q}_{p,i,j,k}= \max \left(QQ.di{r}_{p,i,j,k, forward},QQ.di{r}_{p,i,j,k, reverse}\right)\hfill \\ {}{D}_{i,j,k}= \max \left(D.di{r}_{i,j,k, forward},D.di{r}_{i,j,k, reverse}\right)\hfill \end{array} $$

Thus, they retain the distance terms corresponding to the read direction *d* (forward/reverse) having the largest difference between the nucleotide *k* and the “per position *j*” recalibrated PHRED quality quantile scores. Hence, as read directionality is taken into account, SNV calling using the QQ-SNV model can be performed for all variants present at least once in both forward and reverse direction.

#### Training dataset for classifier model selection and parameter estimation

##### Resampling scheme for the generation of the training data

To generate the training dataset of 96 *in silico* HIV plasmid mixtures, 96 (=5! – 4!) plasmid mixture re-samplings were generated ten times for each of the permutations of plasmid percentages at the ratio of 63, 30, 5, 1.5, and 0.5 %. Note that for plasmid 2 the highest percentage (63 %) could not be assigned because of the lower number of available reads. The ten repeats per permutation were then combined to have a larger coverage depth for training the QQ-SNV model. Moreover, to also have a balanced training dataset in terms of coverage, the coverage depth at each nucleotide position in the mapping region 2253-3749 of the 96 plasmid mixture training samples was chosen to be N = 50,000 reads. Thereto, the re-samplings in the ground set of 960 (96 × 10) plasmid mixtures were guided by filling up each row in the read mapping with reads from the same plasmid, starting at a nucleotide position with the same sampled offset position (0–50). Consequently N = 5000 reads were multinomially distributed over the five plasmids with probabilities cf a given permutation, covering the whole region 2253–3749. Next, to allow for additional errors the bases were recalled and the alignment steps were performed separately for the 960 read sets, each containing a selection of ~150,000 reads (12.7 %) from the original FASTQ/1 dataset. This implies that for each of the read sets first the Illumina Cluster Intensity Files (CIFs) were recreated, retaining the intensities of the subset of reads from the respective re-sampling. Second the basecalling was executed with the Bustard Off-Line Basecaller. Third, the read set was mapped to the HXB2 reference sequence (nt 2203-3799) using CLC Genomics Workbench (CLC bio, Aarhus, Denmark) using the default alignment settings.

##### QQ-SNV classifier model selection and parameter estimation

Training of the QQ-SNV classifier model was performed on 96 *in silico* HIV plasmid mixture data samples (see section Training data), resulting in the following binary logistic regression model with as outcome Y_*in silico*_ = 0 (no error) or 1 (error):

$$ logit\left(\mathrm{Probability}\ \mathrm{of}\ {\mathrm{Y}}_{in\; silico}=0\right)={\beta}_0+{\beta}_1\times QQnor{m}_{5^{th}}+{\beta}_2\times QQnor{m}_{25^{th}}+{\beta}_3\times QQnor{m}_{95^{th}}+{\beta}_4\times Q{Q}_{80^{th}}+{\beta}_5\times D $$

where *Y*_*in silico i*,*j*,*k*_ = *I*{nucleotide *k* at position *j* of sample *i* is a sequencing error}.

In a stepwise regression procedure, using a significance level (*p*-value threshold) of 5E-4 for entering or removing of covariates, the following five quality score derived predictors were selected for the QQ-SNV model: $QQnor{m}_{5^{th}}$, $QQnor{m}_{25^{th}}$, $QQnor{m}_{95^{th}}$, $Q{Q}_{80^{th}}$, and *D*. To increase the sensitivity of the QQ-SNV model when classifying variants, having a predicted SNV probability above 0.5, as being *true*, the QQ-SNV model coefficients (Table 2) were fitted using weighted binary logistic regression to correct for the imbalances in the training data (less SNVs than errors), with weighting factors of 25 and 1 in case of “no sequencing error” and sequencing error, respectively. These weighting factors for rescaling of the probabilities for a SNV probability threshold of 0.5 were chosen to match the false positive : false negative ratio of the unweighted trained model as used to select the quality score derived predictors using as cutoff 0.008 (=12/1497 = the percentage of SNV positions in training region) (Additional file 1: Figure S14).

**Table 2:** QQ-SNV model trained on 96 *in silico* HIV plasmid mixture data samples

Parameter	Coefficient	Significance (P-value)
Intercept	−117.66 (*β*_0_)	<2e-16
$QQnor{m}_{5^{th}}$	4.38 (*β*_1_)	<2e-16
$QQnor{m}_{25^{th}}$	7.37 (*β*_2_)	<2e-16
$QQnor{m}_{95^{th}}$	116.78 (*β*_3_)	<2e-16
$Q{Q}_{80^{th}}$	−3.06 (*β*_4_)	<2e-16
*D*	−0.70 (*β*_5_)	<2e-16

#### Running QQ-SNV

**Fig. 1:**
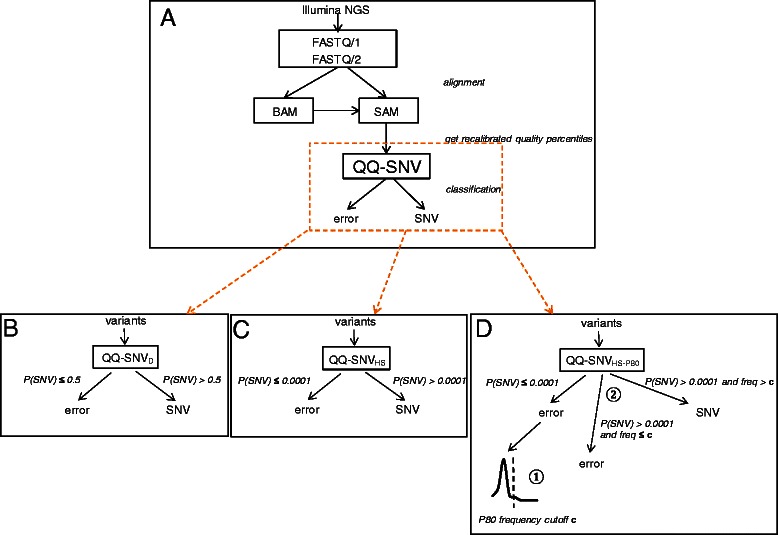
QQ-SNV variant calling. Schematic view of QQ-SNV variant calling methodology starting from Illumina NGS data. **a** QQ-SNV workflow **b** QQ-SNV_D_ **c** QQ-SNV_HS_ **d** QQ-SNV_HS-P80_, 80^th^ percentile (P80) of the QQ-SNV_HS_ distribution of “error” frequencies was used as frequency cutoff to decrease the number of false positives obtained with QQ-SNV_HS_

We have developed a workflow to perform QQ-SNV classification on a new dataset (Fig. 1a). First, an alignment containing the reference mapped FASTQ/1 and/or FASTQ/2 reads needs to be exported as a SAM file [23]. In case the alignment has been saved as a BAM file (binary format) instead, a conversion to the SAM format can be made using samtools [23]. Second, the quality scores of the nucleotides in the reads are to be recalibrated using the quality score recalibration model, and the nucleotide recalibrated quality percentiles and distance metrics, needed in the QQ-SNV model, are to be calculated per aligned nucleotide position and direction of the reads (forward/reverse). Finally, the QQ-SNV classifier model is used for the SNV calling, classifying a variant as SNV (*true* variant) – if the QQ-SNV model predicted SNV probability is above a threshold value of 0.5 (QQ-SNV_D (default)_) – or as a sequencing error (Fig. 1b). While in the training of the QQ-SNV model we rescaled the SNV probabilities for a probability cutoff of 0.5 (see previous section), when applying logistic regression on multiple different datasets the optimal classification threshold on these datasets is rarely exactly 0.5 [25]. Therefore, to increase the sensitivity of the QQ-SNV classifier in detecting low frequency variants, for QQ-SNV_HS (high sensitivity)_ the model coefficients as shown in Table 2 were still used, but now using a SNV probability threshold of 0.0001 (Fig. 1c). Thus, in order to obtain high sensitivity on new data this high sensitivity threshold is taken 15 times lower than the lowest SNV probability obtained for a *true* variant in the ground set of 960 samples, which was 0.00153. However, to avoid the occurrence of many false positives by taking a much lower SNV probability threshold than default, we calculated in addition the 50^th^/75^th^/80^th^/85^th^/90^th^/95^th^ percentile of the QQ-SNV_HS_ distribution (frequency cutoff **c**) of “error” (variants with P(SNV) ≤ 0.0001) frequencies. To reduce false positives, “SNVs” (variants with P(SNV) > 0.0001) are overruled when their frequency is lower than **c** (Fig. 1d). The specificity of QQ-SNV_HS_ will increase when choosing a higher percentile to calculate the frequency threshold. When evaluating QQ-SNV_HS_ using the above different percentiles on the test sets (see section Test data), using the 80^th^ percentile (P80) as frequency cutoff was overall most accurate (see section Results). Therefore, we suggest to use QQ-SNV_HS-P80_ on new datasets.

To run QQ-SNV on the different datasets as analyzed in this paper, we wrote a perl script QQ-SNV_workflow.pl (Additional file 4). Within the QQ-SNV workflow, SAS 9.2 was used for the SNV calling. The SAS default percentile definition was used for calculation of the quality quantiles and frequency thresholds.

#### Parameter settings of competing methods

##### ShoRAH

ShoRAH v0.8 was run. The largest window size was used that worked with the software, corresponding to the sequencing read length. For the other parameters, the default settings were used.

##### V-Phaser 2

V-Phaser 2 v2.0 was run using the default settings, applying strand bias test and correction for multiple testing.

##### LoFreq

LoFreq v0.5.0 was run using the default settings.

## Results

### Training performance and method validation of QQ-SNV

Training of QQ-SNV_D_ on the *in silico* HIV plasmid mixture data with coverage N = 50,000 reads (96 samples with different plasmid mixture proportion), sequenced on the Illumina GAIIx platform (51 bp reads), resulted in 99.2 % sensitivity, 99.9 % specificity, and Positive Predictive Value (PPV) of 75 % (Additional file 2: Table S4). The 9 *true* variants not detected were present at the lowest frequency of 0.5 %. In addition, we analyzed the performance of QQ-SNV on the ground set *in silico* HIV plasmid mixture data with coverage N = 5000 reads (960 samples with ten repeats re-sampled for the 96 plasmid mixture proportions as used in the training data) (Additional file 2: Table S5). Here, besides QQ-SNV_D_ we evaluated QQ-SNV_HS_ and QQ-SNV_HS-P50/75/90_ using as frequency threshold the 50^th^/75^th^/90^th^ percentile (P50, P75 and P90) of the frequency distribution of variants called as error by QQ-SNV_HS_. The sensitivity of the methods in detecting the *true* variants was calculated for different intervals on the *true* variant frequencies. For QQ-SNV_D_ both sensitivity and specificity are above 95 %, but the PPV of 19.6 % is low. The latter is due to the *in silico* resampling at a relatively low coverage depth of 5000 reads and the presence of variants at *true* percentages below 0.5 % (Additional file 1: Figure S6). QQ-SNV_HS_ achieves 100 % sensitivity, but the number of false positives is much higher. When applying QQ-SNV_HS-P90_ the number of false positives is clearly reduced in comparison to QQ-SNV_HS_ and the sensitivity in detecting variants with prevalence < 0.5 % is high (99.7 %) (Additional file 2: Table S5), thus validating the extended version of the QQ-SNV_HS_ approach (Fig. 1d). On the test data in the next sections, then in addition the 80^th^, 85^th^ and 95^th^ percentile (P80, P85 and P95) were calculated of the QQ-SNV_HS_ distribution of “error” frequencies. QQ-SNV_HS-P80_ resulted in the best overall performance.

### Performance evaluation of QQ-SNV on HIV plasmid mixture datasets

The results of the performance comparison of the different methods on the HIV plasmid mixture dataset 1 sequenced on the Illumina GAIIx platform (31 bp reads) are given in Table 3, Additional file 1: Figure S15A and Additional file 2: Table S6. The sensitivity in detecting variants ≥ 1 % is similar for the different methods, except for V-Phaser 2. However, the latter method was equally sensitive as QQ-SNV_HS-P80_ in detecting SNVs at 0.1 %. Note that QQ-SNV_HS-P80_ was also more robust than QQ-SNV_D_ in detecting variants at percentages ≥ 10 %. By overruling 92 % of the QQ-SNV_HS_ false positives, the specificity of QQ-SNV_HS-P80_ is similar as for the existing methods. The computationally most efficient method was QQ-SNV (14 m), followed by V-Phaser 2 (33 m), LoFreq (53 m), and ShoRAH (6h25m).

**Table 3:** Performance on HIV plasmid mixture dataset 1

	QQ-SNV_D_	QQ-SNV_HS-P80_^a^	LoFreq	ShoRAH	V-Phaser 2
Variant %	sens.^b^	sens.^b^	sens.^b^	sens.^b^	sens.^b^
88.889 %	57/61	60/61	59/61	56/61	20/61
10 %	44/50	49/50	47/50	45/50	13/50
1 %	28/34	30/34	23/34	26/34	10/34
0.1 %	2/42	20/42	0/42	7/42	22/42
0.01 %	0/40	1/40	0/40	3/40	8/40
0.001 %	0/30	0/30	0/30	0/30	0/30
	FP^c^	FP^c^	FP^c^	FP^c^	FP^c^
	2	4	0	6	2
	time^de^	time^de^	time^df^	time^dg^	time^dg^
	14m11s	14m11s	53m24s	6h25m	33m5s

^a^80^th^ percentile of the QQ-SNV_HS_ distribution of “error” frequencies was used as frequency cutoff to decrease the number of false positives obtained with QQ-SNV_HS_

^b^sens. is the percentage of *true* variants that were correctly called as SNV

^c^FP is the number of variants incorrectly called as SNV

^d^computation time in hours (h), minutes (m) and seconds (s)

^e^Windows 7 64 bit, 8GB RAM, 3.2GHz

^f^Linux Ubuntu 12.04.4, 57.6 GB RAM, one core (2.3GHz) used

^g^Linux Ubuntu 12.04.4, 57.6 GB RAM, 8 cores (2.3GHz) used in parallel

The results of the performance comparison of the different methods on the HIV plasmid mixture dataset 2 sequenced on the Illumina HiSeq 2000 platform (50 bp reads) are given in Table 4, Additional file 1: Figure S15B and Additional file 2: Table S7. V-Phaser 2 failed on this dataset. Although ShoRAH was the fastest method in producing results, too many variants at high and low frequencies were not detected. Thus QQ-SNV and LoFreq remain for the comparison. QQ-SNV_HS-P80_ was more sensitive than LoFreq at 1.0 % (100 *vs.* 90 %) and at 0.1 % (60 *vs.* 20 %). For QQ-SNV_HS-P80_ one false positive was returned which was not eliminated when applying a P95 frequency threshold (Additional file 2: Table S7), while for LoFreq there were no false positives. On this dataset QQ-SNV was twenty times faster than LoFreq (2h29m *vs.* 2d4h11m). This confirms that LoFreq is slow in detecting variants at high frequency, whereas for QQ-SNV the runtime is entirely due to the high coverage depth (>1 million reads).

**Table 4:** Performance on HIV plasmid mixture dataset 2

	QQ-SNV_D_	QQ-SNV_HS-P80_^a^	LoFreq	ShoRAH	V-Phaser 2
Variant %	sens.^b^	sens.^b^	sens.^b^	sens.^b^	sens.^b^
40.0 %	2/2	2/2	2/2	1/2	na^h^
39.9 %	1/1	1/1	1/1	1/1	na^h^
39.5 %	1/1	1/1	1/1	0/1	na^h^
38.9 %	1/1	1/1	1/1	0/1	na^h^
38.4 %	2/2	2/2	2/2	2/2	na^h^
34.4 %	2/2	2/2	2/2	1/2	na^h^
34.0 %	1/1	1/1	1/1	1/1	na^h^
33.5 %	1/1	1/1	1/1	1/1	na^h^
33.4 %	8/8	8/8	8/8	7/8	na^h^
6.6 %	1/1	1/1	1/1	1/1	na^h^
6.1 %	1/1	1/1	1/1	0/1	na^h^
5.1 %	1/1	1/1	1/1	1/1	na^h^
5.0 %	8/8	8/8	8/8	8/8	na^h^
1.0 %	10/10	10/10	9/10	7/10	na^h^
0.6 %	0/1	1/1	1/1	0/1	na^h^
0.5 %	5/8	8/8	8/8	2/8	na^h^
0.1 %	1/10	6/10	2/10	2/10	na^h^
	FP^c^	FP^c^	FP^c^	FP^c^	FP^c^
	0	1	0	3	na^h^
	time^de^	time^de^	time^df^	time^dg^	time^dg^
	2h29m	2h29m	2d4h11m	57m45s	na^h^

^a^80^th^ percentile of the QQ-SNV_HS_ distribution of “error” frequencies was used as frequency cutoff to decrease the number of false positives obtained with QQ-SNV_HS_

^b^sens. is the percentage of *true* variants that were correctly called as SNV

^c^FP is the number of variants incorrectly called as SNV

^d^computation time in days (d), hours (h), minutes (m) and seconds (s)

^e^Windows 7 64 bit, 8GB RAM, 3.2GHz

^f^Linux Ubuntu 12.04.4, 57.6 GB RAM, one core (2.3GHz) used

^g^Linux Ubuntu 12.04.4, 57.6 GB RAM, 8 cores (2.3GHz) used in parallel

^h^No results could be obtained for the V-Phaser 2 algorithm due to failure of the V-Phaser 2 software tool on our server

### Performance evaluation on HCV plasmid mixture datasets

The results of the performance comparison of the different methods on the HCV plasmid mixture datasets sequenced on the Illumina GAIIx platform (70 bp reads) are given in Table 5, Additional file 1: Figures S15C-D and Additional file 2: Table S8. Similarly as on HIV plasmid mixture dataset 1, for V-Phaser 2 the sensitivity in detecting variants at higher percentages is low. Both QQ-SNV_HS_ and LoFreq were the only methods with 100 % sensitivity for the reads 1, reads 2, and combined reads 1 and reads 2 data for *true* variant frequencies ≥ 1 %. While QQ-SNV_HS_ detects all variants but one (reads 2 at 0.5 %), the number of false positives on reads 1 data is higher than for the other methods. On the reads 1 data, best performance was seen for ShoRAH. When applying a P80 frequency threshold on the QQ-SNV_HS_ SNVs, similar specificity as LoFreq was obtained on the reads 1 data. On the combined reads 1 and reads 2 data only QQ-SNV_HS-P75_ and QQ-SNV_HS-P80_ achieved an accuracy of 100 % (Additional file 2: Table S8) at all *true* variant frequencies. As seen from the Receiver Operating Characteristic (ROC) curves at different coverage depth (Additional file 1: Figures S16-S19), the performance of QQ-SNV is best at the highest sequencing depth and becomes worse for non-UDS datasets (Additional file 2: Table S9). The same is observed for the other methods, although for ShoRAH we note better performance at 10 % downsampling than at 40 % downsampling or for the full HCV plasmid mixture datasets (all paired-end reads) (Additional file 2: Table S9 and Table 5).

**Table 5:** Performance on HCV plasmid mixture datasets

		QQ-SNV_D_		QQ-SNV_HS-P80_^a^		LoFreq		ShoRAH		V-Phaser 2	
Variant %	Pair	sens.^b^	FP^c^	sens.^b^	FP^c^	sens.^b^	FP^c^	sens.^b^	FP^c^	sens.^b^	FP^c^
0.50 %	1^d^	5/5	10	5/5	38	5/5	36	5/5	2	5/5	11
0.50 %	2^e^	0/5	0	0/5	0	0/5	0	0/5	0	3/5	15
0.50 %	1 + 2^f^	1/5	0	5/5	0	2/5	5	2/5	7	3/5	32
1 %	1^d^	5/5	11	5/5	53	5/5	62	5/5	2	5/5	13
1 %	2^e^	1/5	0	5/5	0	5/5	1	2/5	1	2/5	28
1 %	1 + 2^f^	3/5	1	5/5	0	5/5	7	4/5	1	3/5	45
2 %	1^d^	5/5	13	5/5	64	5/5	72	5/5	4	2/5	20
2 %	2^e^	3/5	0	5/5	0	5/5	1	3/5	0	3/5	14
2 %	1 + 2^f^	5/5	0	5/5	0	5/5	7	5/5	2	3/5	28
10 %	1^d^	5/5	6	5/5	42	5/5	75	5/5	0	3/5	10
10 %	2^e^	5/5	0	5/5	0	5/5	0	5/5	0	2/5	28
10 %	1 + 2^f^	5/5	0	5/5	0	5/5	8	5/5	1	2/5	56
		time^gh^		time^gh^		time^gi^		time^gj^		time^gj^	
0.50 %	1^d^	2m27s		2m27s		1m30s		1h45m		8m7s	
0.50 %	2^e^	2m18s		2m18s		1m33s		9h29m		22m3s	
0.50 %	1 + 2^f^	4m42s		4m42s		3m36s		15h4m		2h31m	
1 %	1^d^	2m56s		2m56s		2m27s		1h38m		10m51s	
1 %	2^e^	3m1s		3m1s		2m6s		8h46m		46m44s	
1 %	1 + 2^f^	5m48s		5m48s		5m56s		13h13m		3h46m	
2 %	1^d^	2m35s		2m35s		2m6s		1h34m		10m25s	
2 %	2^e^	2m32s		2m32s		1m53s		8h50m		41m1s	
2 %	1 + 2^f^	4m59s		4m59s		4m34s		14h56m		1h47m	
10 %	1^d^	3m5s		3m5s		4m26s		1h46m		13m45s	
10 %	2^e^	2m30s		2m30s		2m35s		14h13m		33m24s	
10 %	1 + 2^f^	5m29s		5m29s		9m53s		7h57m		1h47m	

^a^80^th^ percentile of the QQ-SNV_HS_ distribution of “error” frequencies was used as frequency cutoff to decrease the number of false positives obtained with QQ-SNV_HS_

^b^sens. is the percentage of *true* variants that were correctly called as SNV

^c^FP is the number of variants incorrectly called as SNV

^d^Reads 1 of paired-end reads

^e^Reads 2 of paired-end reads (sequenced after paired-end turn)

^f^All paired-end reads

^g^computation time in hours (h), minutes (m) and seconds (s)

^h^Windows 7 64 bit, 8GB RAM, 3.2GHz

^i^Linux Ubuntu 12.04.4, 57.6 GB RAM, one core (2.3GHz) used

^j^Linux Ubuntu 12.04.4, 57.6 GB RAM, 8 cores (2.3GHz) used in parallel

#### Paired-end vs. single-end data

Although, on these HCV data sets QQ-SNV_HS-P80_ obtained perfect performance on the combined reads 1 and reads 2 data, on the reads 1 data QQ-SNV_D_ is more accurate than QQ-SNV_HS-P80_.

#### Computational efficiency

**Fig. 2:**
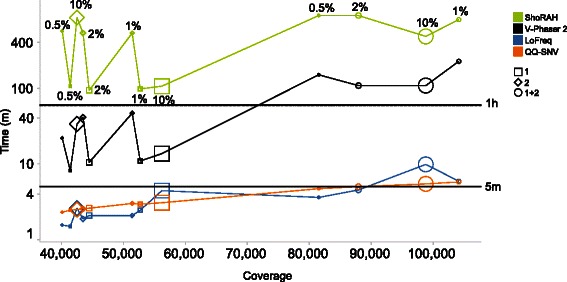
QQ-SNV *vs.* other methods: computational efficiency on HCV plasmid mixture datasets. On the x-axis is the coverage, which is the average number of reads per position. On the y-axis is the computation time in minutes. A reference line is shown at 5 and 60 min. The size of the data points (reads 1, reads 2 and reads 1 + 2) shown for the different HCV datasets corresponds to the variant percentage (0.5 %, 1 %, 2 % and 10 %)

For QQ-SNV the runtime needed increased linearly with coverage and was independent of variant frequency (Fig. 2 and Table 5), whereas for LoFreq computational efficiency decreased for detection of variants at 10 %. At a coverage depth of 98,800 reads for the combined reads 1 and reads 2 data QQ-SNV was 1.8 times faster than LoFreq (5m29s *vs.* 9m53s). V-Phaser 2 and ShoRAH needed considerably more time: 1h47m and 7h57m, respectively. For detection of variants ≤ 2 % computational efficiency of QQ-SNV was similar as for LoFreq (Fig. 2 and Table 5).

### Performance evaluation on H1N1 clinical dataset

The results of the performance comparison of the different methods on a H1N1 clinical sample, sequenced on Illumina GAIIx and MiSeq platforms (37 bp reads) are given in Table 6 and Additional file 2: Table S10. Here, variants in the region 405–425 of the neuraminidase gene were all present at a very low frequency below 0.5 %. In [12], four SNVs were called at the positions 410, 413, 415, and 421, and the other seventeen positions were considered “non-variant”.

**Table 6:** Performance on H1N1 clinical sample

		QQ-SNV_D_		QQ-SNV_HS-P80_^a^		LoFreq		ShoRAH		V-Phaser 2	
Illumina sequencer	Pair	sens.^b^	FP^c^	sens.^b^	FP^c^	sens.^b^	FP^c^	sens.^b^	FP^c^	sens.^b^	FP^c^
GAIIx	1^d^	0/4	0	4/4	2	2/4	0	0/4	0	1/4	0
GAIIx	2^e^	0/4	0	4/4	1	3/4	0	0/4	0	0/4	0
GAIIx	1 + 2^f^	0/4	0	4/4	4	3/4	0	0/4	0	na^k^	na^k^
MiSeq	1^d^	2/4	1	4/4	5	0/4	0	0/4	0	0/4	0
MiSeq	2^e^	2/4	0	4/4	2	0/4	0	na^k^	na^k^	0/4	0
MiSeq	1 + 2^f^	4/4	3	4/4	6	0/4	0	na^k^	na^k^	na^k^	na^k^
		time^gh^		time^gh^		time^gi^		time^gj^		time^gj^	
GAIIx	1^d^	12m51s		12m51s		7m46s		6h1m		43m15s	
GAIIx	2^e^	13m5s		13m5s		7m55s		6h41m		43m6s	
GAIIx	1 + 2^f^	21m56s		21m56s		19m1s		2h18m		na^k^	
MiSeq	1^d^	1m11s		1m11s		26 s		5h46m		3m40s	
MiSeq	2^e^	1m23s		1m23s		24 s		na^k^		3m55s	
MiSeq	1 + 2^f^	2m45s		2m45s		1 m		na^k^		na^k^	

^a^80^th^ percentile of the QQ-SNV_HS_ distribution of “error” frequencies was used as frequency cutoff to decrease the number of false positives obtained with QQ-SNV_HS_

^b^sens. is the percentage of SNVs as identified in [12] that were called as SNV by QQ-SNV in region 405–425 of NA gene in H1N1 BN3 sample

^c^FP is the number of “non-variants” in [12] that were called as SNV by QQ-SNV

^d^Reads 1 of paired-end reads

^e^Reads 2 of paired-end reads (sequenced after paired-end turn)

^f^All paired-end reads

^g^computation time in hours (h), minutes (m) and seconds (s)

^h^Windows 7 64 bit, 8GB RAM, 3.2GHz

^i^Linux Ubuntu 12.04.4, 57.6 GB RAM, one core (2.3GHz) used

^j^Linux Ubuntu 12.04.4, 57.6 GB RAM, 8 cores (2.3GHz) used in parallel

^k^No result could be obtained for the ShoRAH/V-Phaser 2 algorithm due to failure of the ShoRAH/V-Phaser 2 software tool on our server

#### Paired-end vs. single-end data

From the GAIIx data, QQ-SNV_D_ did not detect any of these four SNVs (Table 6). From the MiSeq reads 1 or reads 2 data, 2/4 of these SNVs could be detected by QQ-SNV_D_. Only for the MiSeq combined reads 1 and reads 2 data QQ-SNV_D_ had 100 % sensitivity in detecting these four SNVs, at a specificity of 82.4 % (percentage of “non-variants” in [12], also classified as “non-variant” by QQ-SNV) (Table 6). In contrast, QQ-SNV_HS_ and QQ-SNV_HS-P50/P75/P80/P85/P90/P95_ was 100 % sensitive when presented with either GAIIx/MiSeq reads 1 data, reads 2 data, or the combined reads 1 and reads 2 data (Table 6 and Additional file 2: Table S10). Due to failure, paired-end results could not be obtained for V-Phaser 2 on both the GAIIx and MiSeq data and for ShoRAH on the MiSeq data.

#### Effect on different Illumina sequencer types

Whereas, the four SNVs were consistently detected from both GAIIx and MiSeq data using QQ-SNV_HS_ or QQ-SNV_HS_ applying a SNV frequency threshold (Table 6 and Additional file 2: Table S10), none of the SNVs were detected from the other methods on the MiSeq data (Table 6). However, V-Phaser 2 and LoFreq were able to detect one and three SNVs from the GAIIx data, respectively. ShoRAH did also not detect any of the SNVs from the GAIIx data (Table 6).

When comparing the specificity of QQ-SNV_HS-P80_ for the two platforms, a higher specificity was obtained from GAIIx versus MiSeq data: 76.5–94.1 % *vs.* 64.7–88.2 %. Table 7 lists the different positions where a nucleotide variant was classified as SNV by QQ-SNV_HS-P80_ from the combined reads 1 and reads 2 data for GAIIx and MiSeq. For the GAIIx data, the four SNVs considered to be *true* were detected in the frequency range [0.25–0.35 %], whereas the variants called as SNV by QQ-SNV_HS-P80_ only were in the frequency range [0.13–0.18 %]. For the MiSeq data, the four SNVs were detected in the frequency range [0.14–0.28 %], and the variants called as SNV by QQ-SNV_HS-P80_ only were in the frequency range [0.07–0.10 %]. For the MiSeq data, two variants with frequency < 0.1 % had high predicted SNV probabilities 0.882 and 0.999 (Table 7), although they were not called as SNV on the GAIIx. While a reason could be that a higher coverage was used on the GAIIx compared to MiSeq, it might indicate that the lower limit of reliable detection was exceeded.

**Table 7:** SNVs called by QQ-SNV_HS-P80_ on H1N1 clinical sample (all paired-end reads)

	GAIIx		MiSeq		SNV identified in [12]
Position^a^	Percentage^b^	P(SNV)^c^	Percentage^b^	P(SNV)^c^	
406	na^d^	na^d^	0.09 %	0.99923	No
409	0.13 %	0.00110	0.10 %	0.00517	No
410	0.35 %	0.05514	0.28 %	0.60047	Yes
411	0.13 %	0.00016	0.09 %	0.00318	No
413	0.26 %	0.00482	0.14 %	0.98815	Yes
415	0.25 %	0.00207	0.16 %	0.98665	Yes
418	0.18 %	0.00011	0.09 %	0.00769	No
421	0.26 %	0.04058	0.16 %	0.94675	Yes
423	0.15 %	0.00012	0.08 %	0.02083	No
425	na^d^	na^d^	0.07 %	0.88219	No

^a^Nucleotide position in neuraminidase (NA) gene

^b^Observed frequency of single nucleotide variant called by QQ-SNV

^c^Probability that variant is a SNV, as predicted by QQ-SNV

^d^Not classified as SNV by QQ-SNV_HS-P80_, P(SNV) ≤ 0.0001

**Fig. 3:**
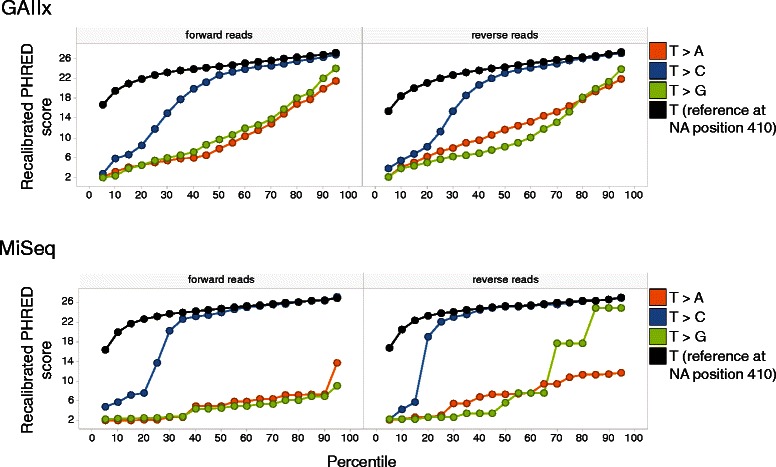
Quality Quantile plot. SNV 410 T > C is called by QQ-SNV_HS-P80_ both on GAIIx and MiSeq for H1N1 clinical sample BN3 [12] at position 410 of the neuraminidase (NA) gene

The Quality Quantile plot for the nucleotides at NA position 410 for the combined reads 1 and reads 2 data, and for both GAIIx and MiSeq is shown in Fig. 3. The distance between the recalibrated quality quantile curves of the nucleotide C and the reference nucleotide T is small enough for QQ-SNV_HS-P80_ to detect the SNV 410 T > C. This distance is smaller for the MiSeq data compared to the GAIIx data, which is reflected in a higher QQ-SNV predicted SNV probability for MiSeq (0.60 *vs.* 0.055). For sequencing errors the curve in forward direction can look very different from the curve reflecting the reverse direction (e.g. 410 T > G for MiSeq).

## Discussion

We described our novel method for SNV calling on NGS data of viral populations, called QQ-SNV, and compared its performance to the existing methods LoFreq, V-Phaser 2 and ShoRAH. Our methodology differs from these other methods in using a logistic regression model. Therefore, QQ-SNV is more related to the regression classifier methods in [16, 18] that were developed for SNV detection in a genome wide and low coverage situation, whereas the goal of our method is to detect *true* variants at low frequency on ultra-deep sequencing datasets. Furthermore, we aimed for a method that can be applied on different Illumina datasets without retraining of the model. Thereto, the QQ-SNV model was trained on a multi-sample dataset of *in silico* plasmid mixtures, without using any sequence context specific covariates for the model. Instead, we introduced “distance metrics” as covariates to compare the nucleotide quality quantiles to the “per position” reference quality. For future research, incremental further improvement of the QQ-SNV model might be possible, e.g. by exploration of other distance metrics in a simulation study or by implementing an extension of our method to enable to call variants at the codon level [26, 27] in addition to SNVs.

In comparison with many of the existing methods, the QQ-SNV method is a computational lightweight variant detection method that limits pre- and post-processing of the data. This results from taking as input the “raw” read mapping data, no filtering or trimming is required. Error-recalibration is incorporated, but QQ-SNV does not perform error-correction (unlike e.g. ShoRAH). Although QQ-SNV returns the “observed” frequency percentages of the *true* variants (thus, biased by the errors), the nucleotide counts are not used inside the method. For QQ-SNV a strand bias test filter is not needed as read directionality is already used in the regression, whereas for e.g. V-Phaser 2 applying a strand bias test led to not detecting SNVs at the higher percentages. Moreover, whereas in a strand bias test one assesses differences in frequency, the novelty of the QQ-SNV method is in evaluating differences in quality instead. For default application of QQ-SNV, using a SNV probability cutoff of 0.5 (QQ-SNV_D_), no filtering needs to be applied after classification. Whereas QQ-SNV_D_ has similar performance as the existing methods, to increase sensitivity a QQ-SNV probability cutoff of 0.0001 was used (QQ-SNV_HS_). Here, to avoid many false positives one frequency based variant filtering step was implemented to overrule SNVs called by QQ-SNV_HS_ when the observed frequency is below the 80^th^ percentile calculated on the QQ-SNV_HS_ distribution of “error” frequencies (QQ-SNV_HS-P80_). On the test sets the results obtained with QQ-SNV_HS-P80_ were consistent for the different Illumina platforms: GAIIx, MiSeq and HiSeq 2000. Although the training of our model has been performed on a fixed read length of 51 bp, QQ-SNV is developed for any read length. Therefore, for verification we performed the QQ-SNV workflow (Fig. 1) on 250 bp sequencing reads of an influenza A plasmid mixture dataset [28]. This confirmed applicability of QQ-SNV for read lengths enabled by the current Illumina sequencers (Additional file 5). Based on the presented results we suggest, regardless of quality of the data obtained or the Illumina platform used for the sequencing, to use QQ-SNV_HS-P80_ to detect *true* variants at low frequency as well as at higher frequencies on the combined reads 1 and reads 2 data (in case of paired-end data), or on reads 1 data (in case no paired end data is available). However, in case specificity is considered more important than sensitivity QQ-SNV_D_ can be applied instead. In case single end data is processed separately and paired end data is also available, our recommendation to use QQ-SNV_D_ *vs.* QQ-SNV_HS-P80_ on the single end data is cf given below. QQ-SNV does not need reference control samples. Yet in case these are available, running QQ-SNV on the controls might give indication on how to more optimally choose the SNV probability cutoff and/or the variant frequency threshold to be used for the clinical samples in the experiment.

Although on the newest Illumina machines quality degradation should no longer be a major issue for reads 2 data of paired-end reads [13], for both the HIV and HCV plasmids data, a lower quality was observed for the reads 2 compared to the reads 1 data. Therefore, it could be valuable in case paired-end sequencing data was obtained not to perform the variant detection on the combined reads 1 and reads 2 data only, but as a quality check also for the reads 1 and/or reads 2 data, separately. When separately processing the reads 1 and/or reads 2 data, paired end information could then also be used in deciding whether to apply QQ-SNV_D_ or QQ-SNV_HS-P80_ for the “final” single-end variant calling, by using the following two rules: I) If the number of variants identified using QQ-SNV_HS-P80_ on the reads 1 and reads 2 data combined is lower than the number of variants identified using QQ-SNV_HS-P80_ on the reads 1 data, then use QQ-SNV_D_ for the final variant calling on the reads 1 data, else use QQ-SNV_HS-P80_. II) If by the above rule QQ-SNV_D_ is used for the final variant calling on the reads 1 data and if the number of variants identified using QQ-SNV_D_ on the reads 2 data is larger than the number of variants identified using QQ-SNV_D_ on the reads 1 data then use QQ-SNV_D_ for the final variant calling on the reads 2 data, else use QQ-SNV_HS-P80_. While we did not notice quality degradation of the reads 2 data for the H1N1 clinical sample, the predicted SNV probabilities were higher for MiSeq than for GAIIx. However, we note that on the MiSeq the experiment was performed with a 10-fold lower coverage depth than on the GAIIx.

## Conclusions

We have developed QQ-SNV for single nucleotide variant detection from NGS deep sequencing data. Different from the existing methods ShoRAH and V-Phaser 2, QQ-SNV is tailor-made for the Illumina NGS systems, a platform expected to be increasingly used for clinical diagnostic purposes. Our focus on the error profile of a single NGS technology, combined with the fact that we trained a regression model on a multi-sample dataset designed to contain *true* variants with percentages populated in the range [0.5-100 %], contributed to the improved performance of QQ-SNV in detecting variants in comparison to the ShoRAH, and V-Phaser 2 algorithms, and in detecting low frequency variants in comparison to LoFreq.

Importantly, instead of modeling nucleotide counts or using any particular sequence specific knowledge, we use the recalibrated quality quantiles, which were calculated per read direction, as the only main parameters in the QQ-SNV model to predict SNV probabilities from the observed quality distributions of the nucleotides present at a particular position in the read mapping. Therefore, in addition to the analysis of viral population data, as exemplified in this manuscript, QQ-SNV could potentially also be applied to any other type of heterogeneous population data.

Finally, QQ-SNV has the advantage of being extremely computationally efficient in handling “ultradeep” read sets, since SNV calling is reduced to a classification method based on logistic regression.

## Availability and requirements

**Project name:** QQ-SNV

**Project home page:** http://sourceforge.net/projects/qqsnv/

**Operating system:** Platform independent

**Programming language:** SAS 9.2, perl

**Other requirements:** Illumina sequencing

**License:** GNU GPL

Any restrictions to use by non-academics: none

## Additional files

Additional file 1:
**Supplementary Figures.** Quality scores Illumina and Quality Quantile plot “raw” (**Figures S1 and S2**). Quality scores and Quality Quantile plot after recalibration (**Figures S3 and S4**). Coverage of plasmids in the training data (**Figure S5**) and distribution of *true* frequencies of *in silico* SNVs in the ground set of 960 samples (**Figure S6**). Distance metrics considered for the QQ-SNV model: QQnorm, QQ, and D (**Figures S7-S9**). ROC curves for QQ-SNV comparing quality score recalibration *vs.* no quality score recalibration on the HCV plasmid mixture test datasets (**Figure S10**). Distance metrics per read direction: QQnorm.dir, QQ.dir, and D.dir (**Figures S11-S13**). Choice of weights for fitting the QQ-SNV model parameters (**Figure S14**). Performance of QQ-SNV_HS-P80_, LoFreq, ShoRAH and V-Phaser 2 on the plasmid mixture test datasets (**Figure S15**). ROC curves for QQ-SNV at different coverage depths (**Figures S16-S19**). (PDF 420 kb)
Additional file 2:
**Supplementary Tables.** Sequence and error profile of HIV plasmids (**Tables S1-S3**). Training performance of QQ-SNV (**Table S4**), performance of QQ-SNV on *in silico* HIV plasmid mixture ground set (**Table S5**) and performance of QQ-SNV on HIV, HCV and H1N1 test sets (**Tables S6-S10**). (PDF 84 kb)
Additional file 3:
**Supporting evidence for stability of coefficients in the QQ-SNV companion logistic regression recalibration model.** (PDF 6 kb)
Additional file 4:
**QQ-SNV.** The ZIP archive contains the following three files: the main perl script file to be executed QQ-SNV_workflow.pl and the SAS files QQ-SNV.sas and get_quantiles.sas. To test QQ-SNV, the read alignment files in SAM format for the HCV plasmid mixture data (downsampled 40 %) and the HCV 1b reference file in FASTA format can be downloaded from http://sourceforge.net/projects/qqsnv. To run QQ-SNV execute the following command: perl QQ-SNV_workflow.pl. (ZIP 5 kb)
Additional file 5:
**Supporting evidence for applicability of QQ-SNV for SNV calling on Illumina NGS sequencing data of any read length.** (PDF 32 kb)
