# Supplementary material for: QQ-SNV: single nucleotide variant detection at low frequency by comparing the quality quantiles
Source: BMC Bioinformatics. 2015 Nov 10;16:379. doi: 10.1186/s12859-015-0812-9 (PMC4641353; doi:10.1186/s12859-015-0812-9)
Supplement: Additional file 1: — Supplementary Figures. Quality scores Illumina and Quality Quantile plot “raw” (Figures S1 and S2). Quality scores and Quality Quantile plot after recalibration (Figures S3 and S4). Coverage of plasmids in the training data (Figure S5) and distribution of true frequencies of in silico SNVs in the ground set of 960 samples (Figure S6). Distance metrics considered for the QQ-SNV model: QQnorm, QQ, and D (Figures S7-S9). ROC curves for QQ-SNV comparing quality score recalibration vs. no quality score recalibration on the HCV plasmid mixture test datasets (Figure S10). Distance metrics per read direction: QQnorm.dir, QQ.dir, and D.dir (Figures S11-S13). Choice of weights for fitting the QQ-SNV model parameters (Figure S14). Performance of QQ-SNVHS-P80, LoFreq, ShoRAH and V-Phaser 2 on the plasmid mixture test datasets (Figure S15). ROC curves for QQ-SNV at different coverage depths (Figures S16-S19). (PDF 420 kb) [file 12859_2015_812_MOESM1_ESM.pdf]

Figure S1. Quality scores Illumina ("raw")

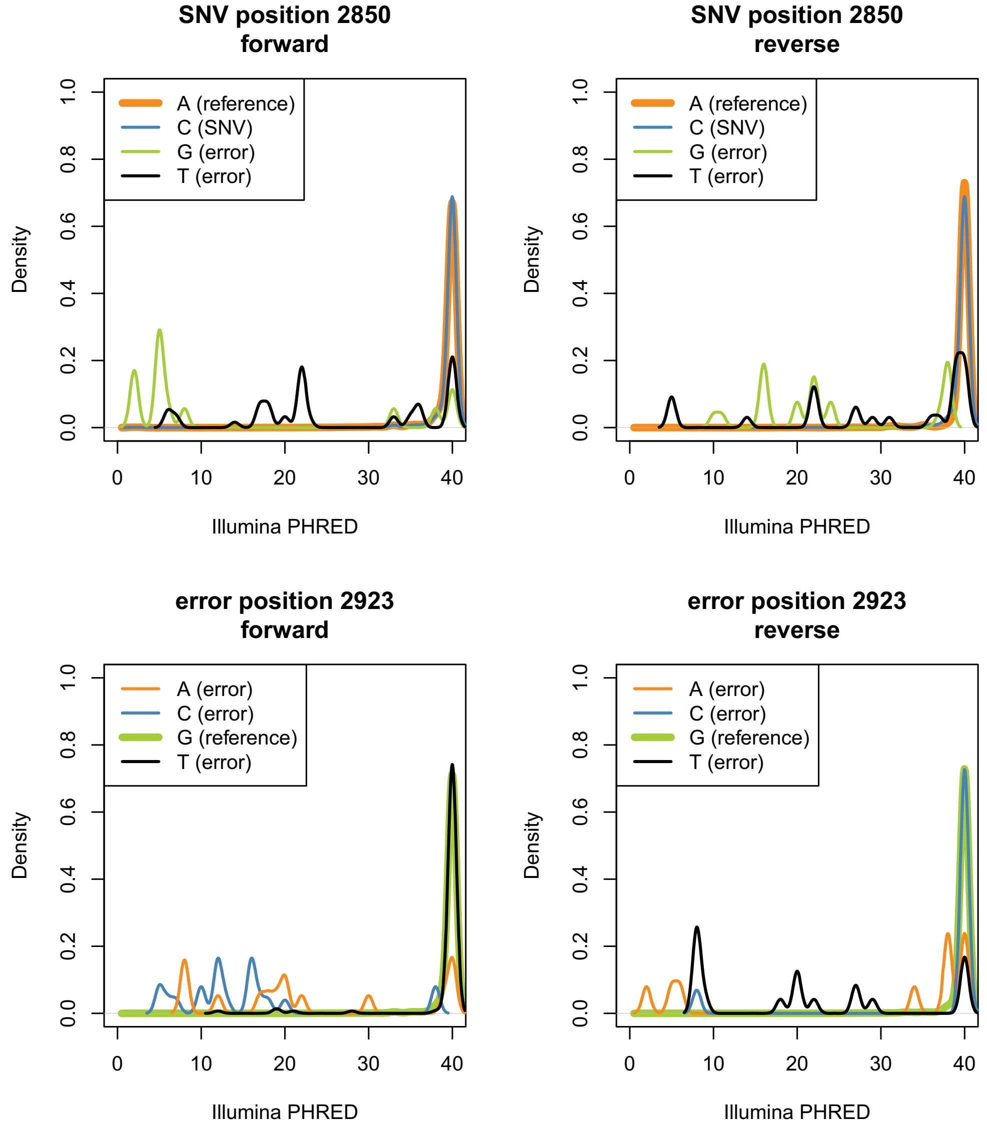

Distributional density of the Illumina PHRED scores per nucleotide (A/C/G/T) in the forward and reverse read mapping at SNV position 2850 and at error position 2923 for training sample 1 (out of 96 *in silico* read sets). The probability density distribution is different for errors compared to "no error" (reference/SNV) in forward and/or reverse read direction.

Figure S2. Quality Quantile plot ("raw")

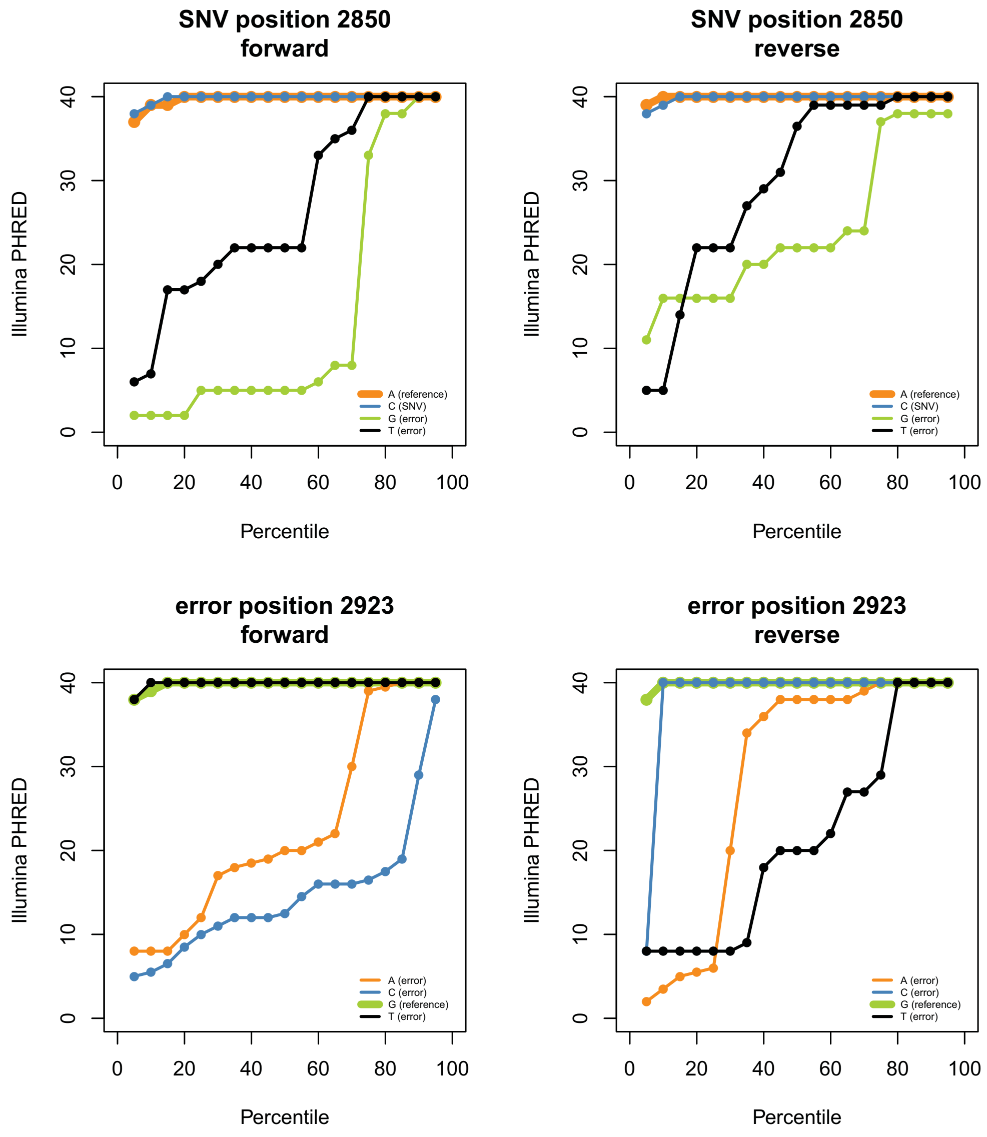

Illumina PHRED scores per nucleotide (A/C/G/T) in the forward and reverse read mapping summarized for nineteen percentiles (5th,10th,...,95th) at SNV position 2850 and at error position 2923 for training sample 1 (out of 96 *in silico* read sets). The cumulative distribution is different for errors compared to "no error" (reference/SNV) in forward and/or reverse read direction.

Figure S3. Quality scores recalibrated

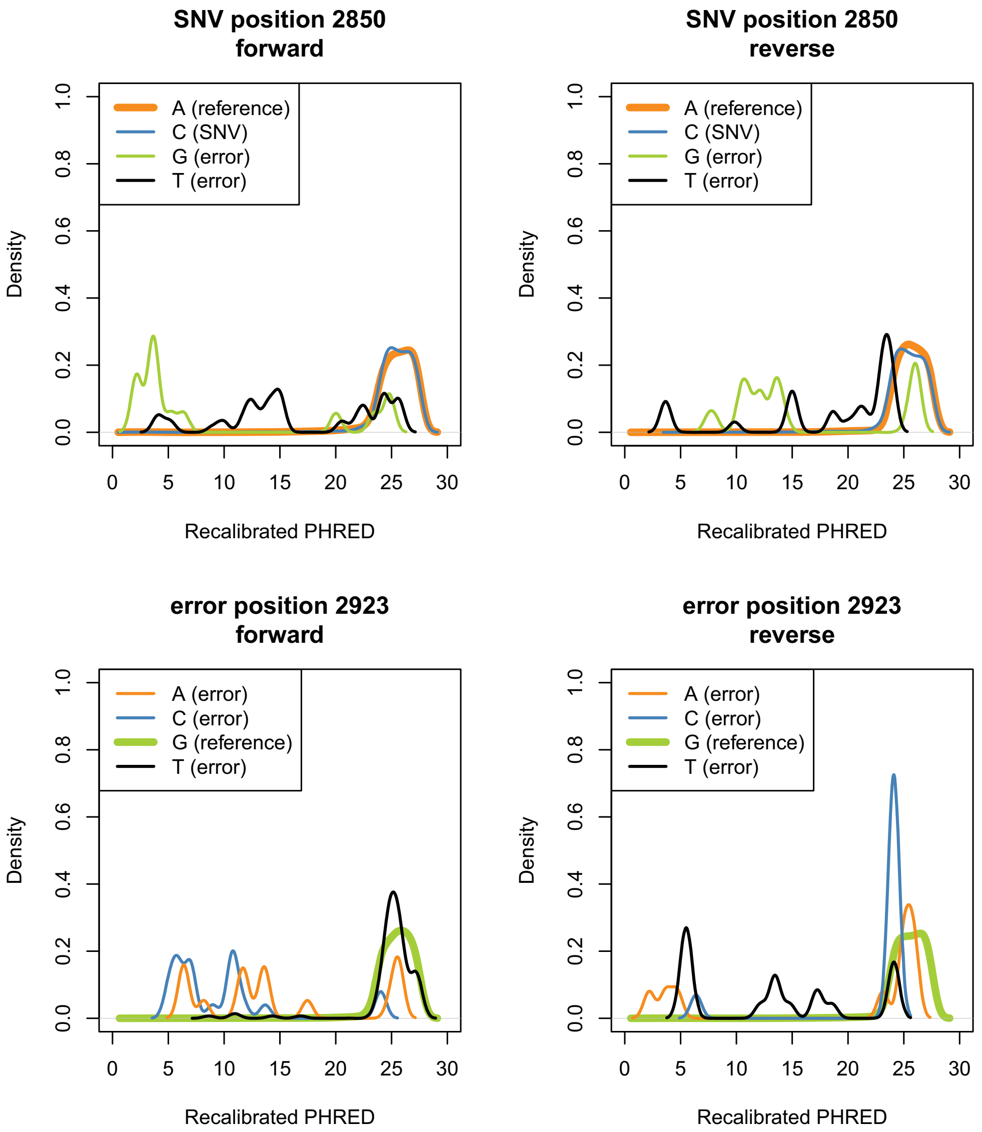

Distributional density of the recalibrated PHRED scores per nucleotide (A/C/G/T) in the forward and reverse read mapping at SNV position 2850 and at error position 2923 for training sample 1 (out of 96 *in silico* read sets). The probability density distribution is different for errors compared to "no error" (reference/SNV) in forward and reverse read direction.

Figure S4. Quality Quantile plot (recalibrated)

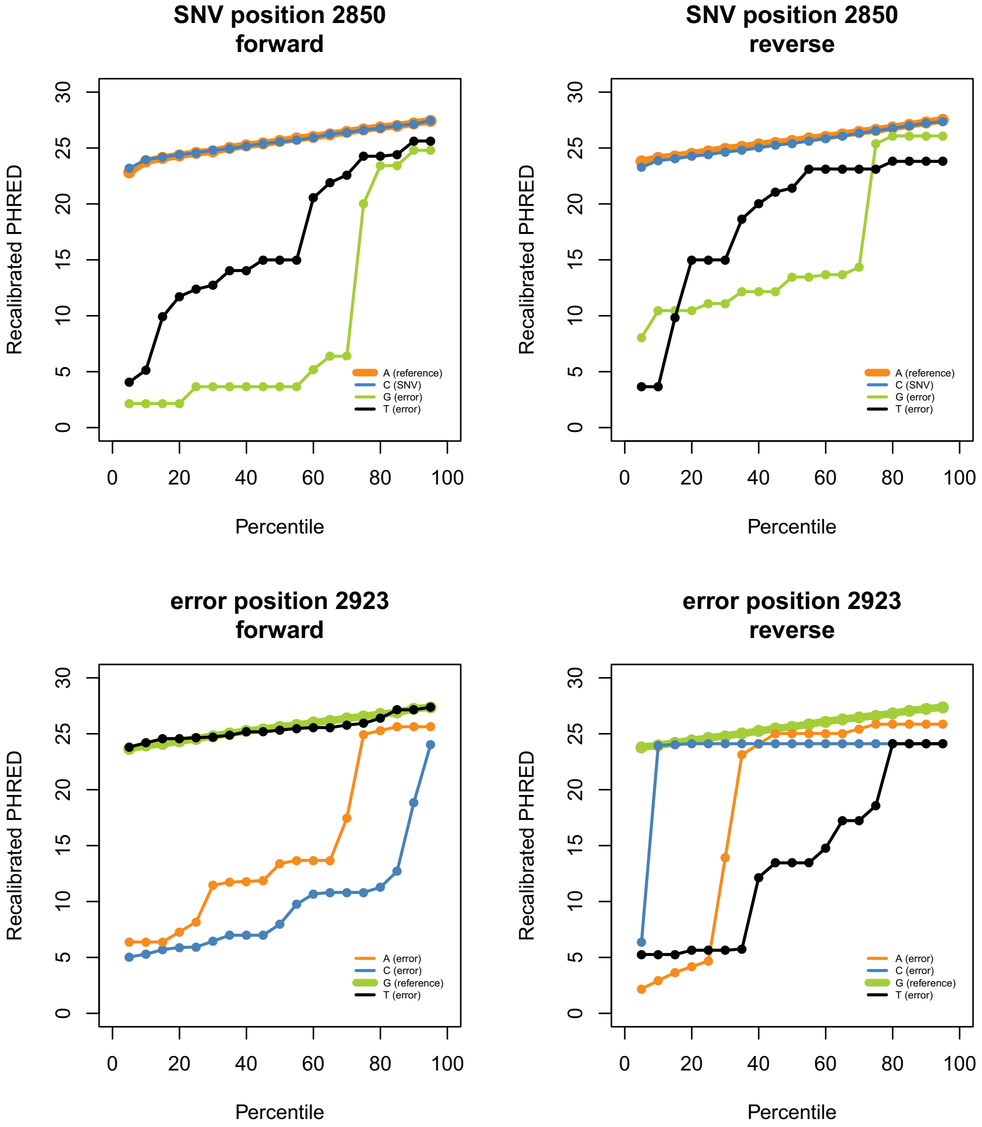

Recalibrated PHRED scores per nucleotide (A/C/G/T) in the forward and reverse read mapping summarized for nineteen percentiles (5th,10th,...,95th) at SNV position 2850 and at error position 2923 for training sample 1 (out of 96 *in silico* read sets). The cumulative distribution is different for errors compared to "no error" (reference/SNV) in forward and reverse read direction.

Figure S5. Coverage of plasmids in FASTQ/1 training data

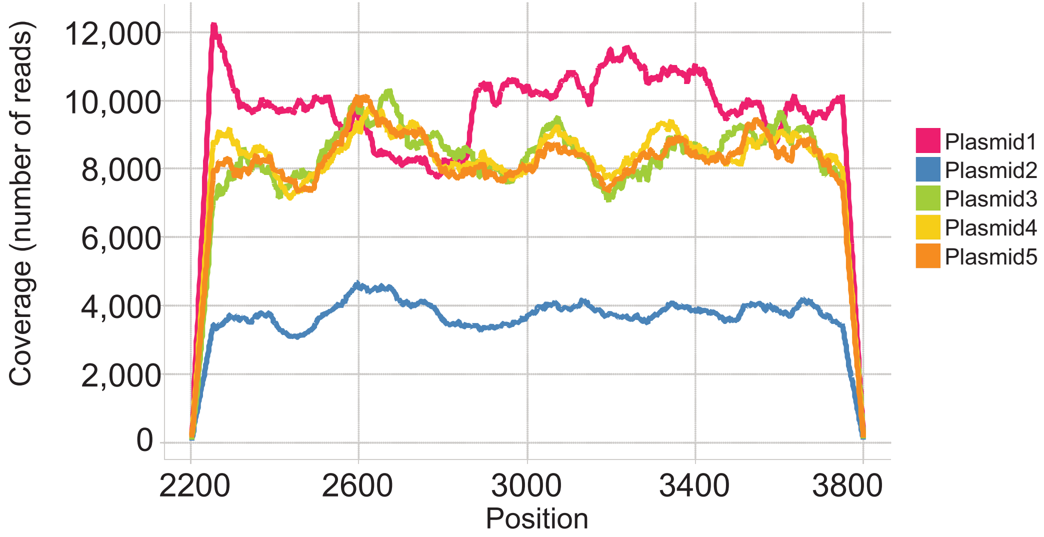

Five HIV−1 plasmids were sequenced with Illumina GAIIx in one lane. The coverage per plasmid derived from a total of 1,183,162 reads, with at least one nucleotide in the PR−RT region from nucleotide position 2253 to 3749, is shown.

Figure S6. SNVs in *in silico* set of 960 samples

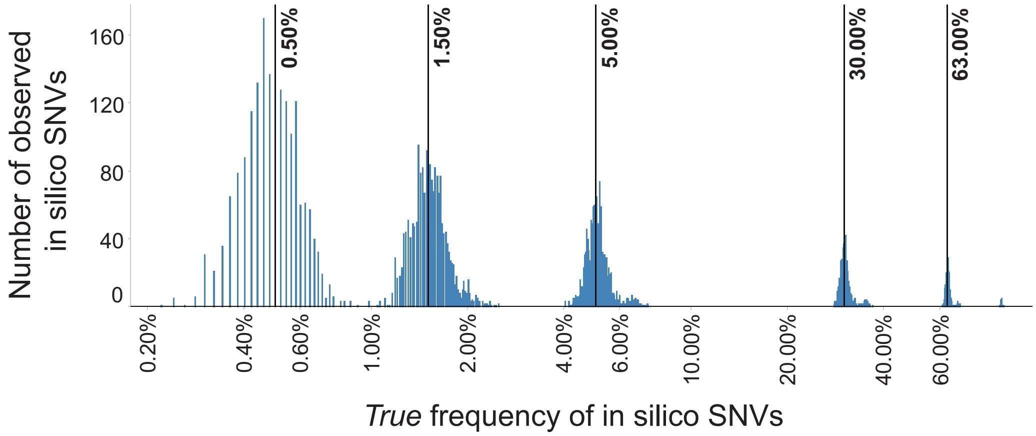

Distribution of so−called *true* frequencies of 9,600 *in silico* SNVs (10 per sample). The 1,920 variants with *true* frequency equal to 100% (always present) at position 2259 and 2927 are not shown. On the y−axis in the histogram are the number of variants with the same *true* frequency sampled around the five chosen frequencies (0.5%, 1.5%, 5%, 30%, and 63%). The *true* frequencies on the x−axis are shown in log_{10} scale.

Figure S7. QQnorm

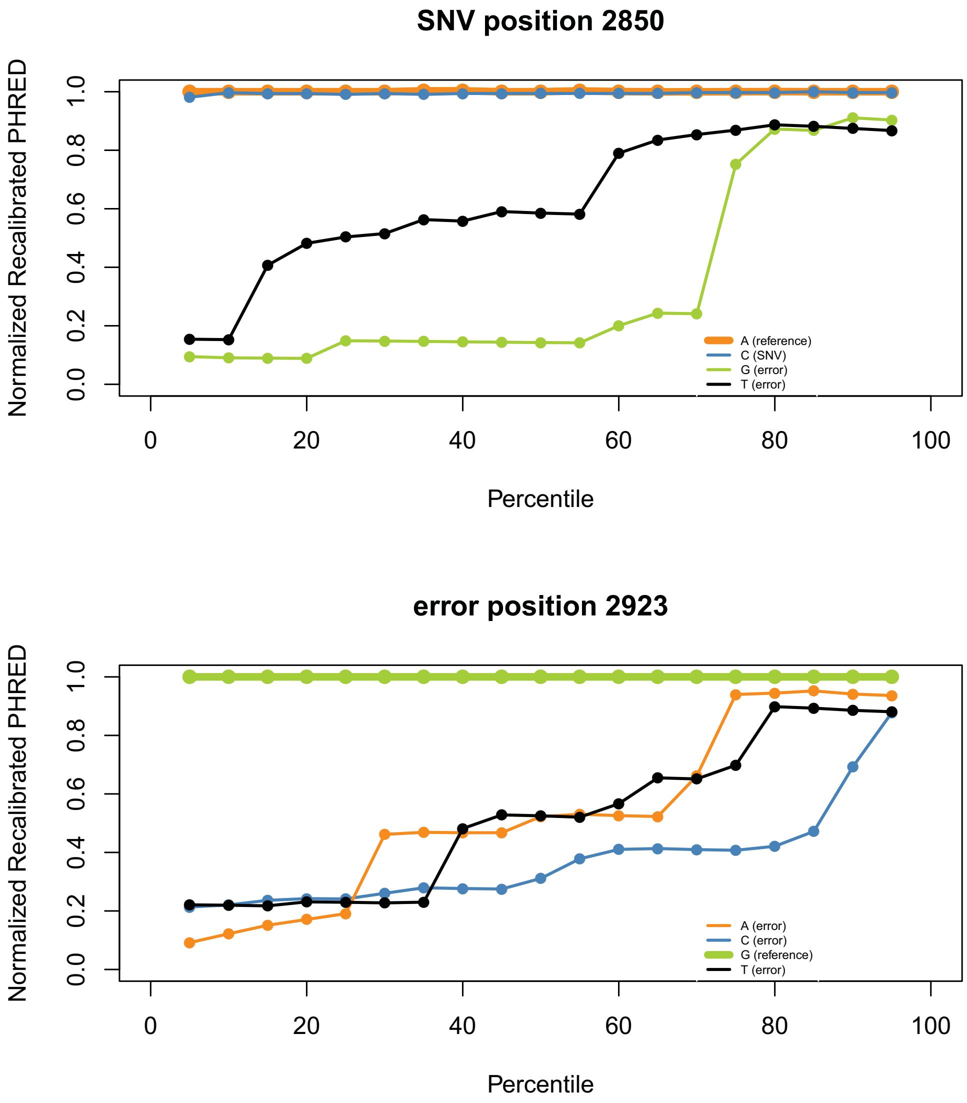

Normalized recalibrated PHRED scores per nucleotide (A/C/G/T) minimized over the read mapping direction (forward/reverse) and calculated for nineteen percentiles (5th,10th,...,95th) at SNV position 2850 and at error position 2923 for training sample 1 (out of 96 *in silico* read sets).

Figure S8. QQ

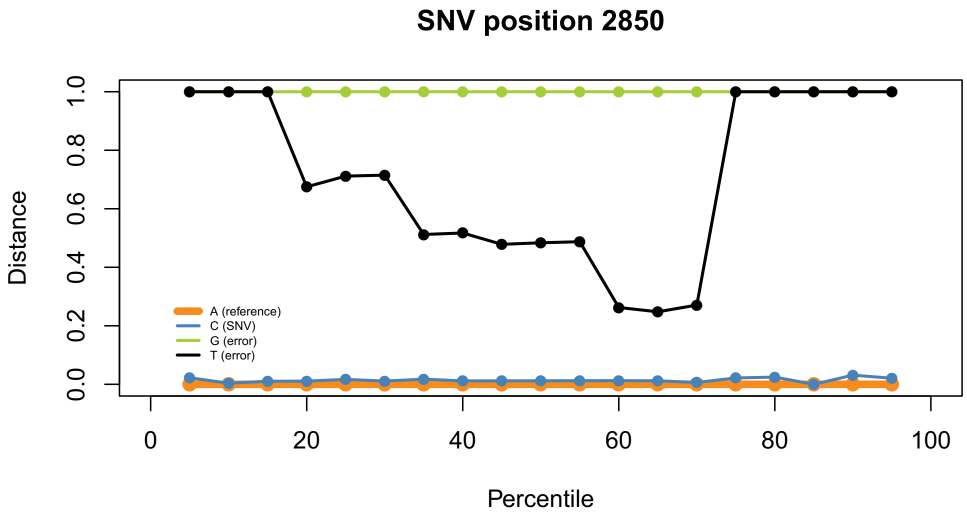

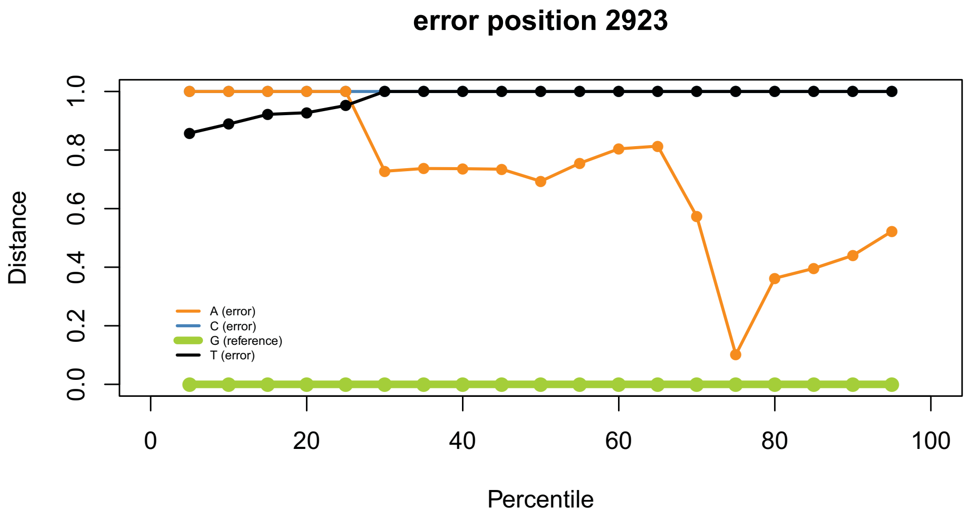

Distance per nucleotide (A/C/G/T) in the range [0,1] maximized over the read mapping direction (forward/reverse) and calculated for nineteen percentiles (5th,10th,...,95th) relative to the nucleotide with worst quality at SNV position 2850 and at error position 2923 for training sample 1 (out of 96 *in silico* read sets).

Figure S9. D

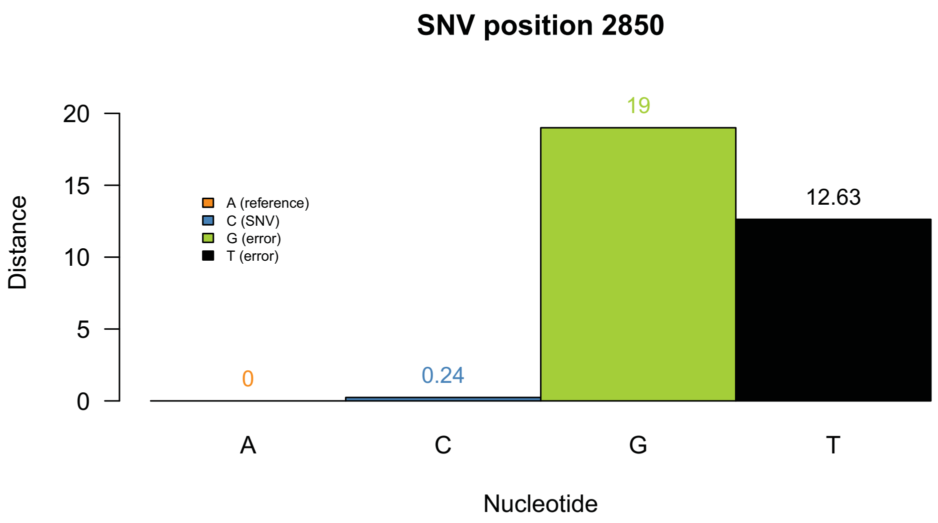

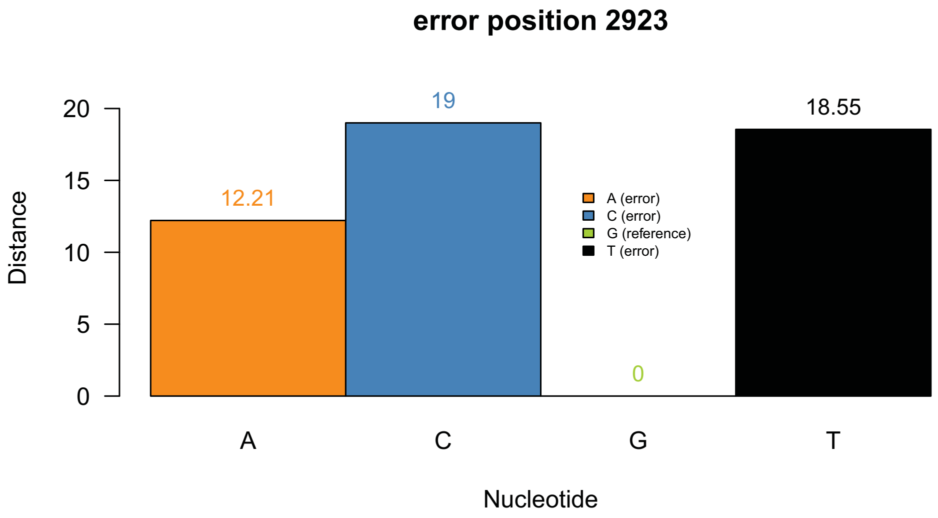

Distance per nucleotide (A/C/G/T) in the range [0,19] maximized over the read mapping direction (forward/reverse) and calculated as the sum of distances for nineteen percentiles (5th,10th,...,95th) relative to the nucleotide with worst quality at SNV position 2850 and at error position 2923 for training sample 1 (out of 96 *in silico* read sets).

Figure S10.
ROC curves for QQ−SNV without variant frequency filtering on HCV plasmid mixture test datasets

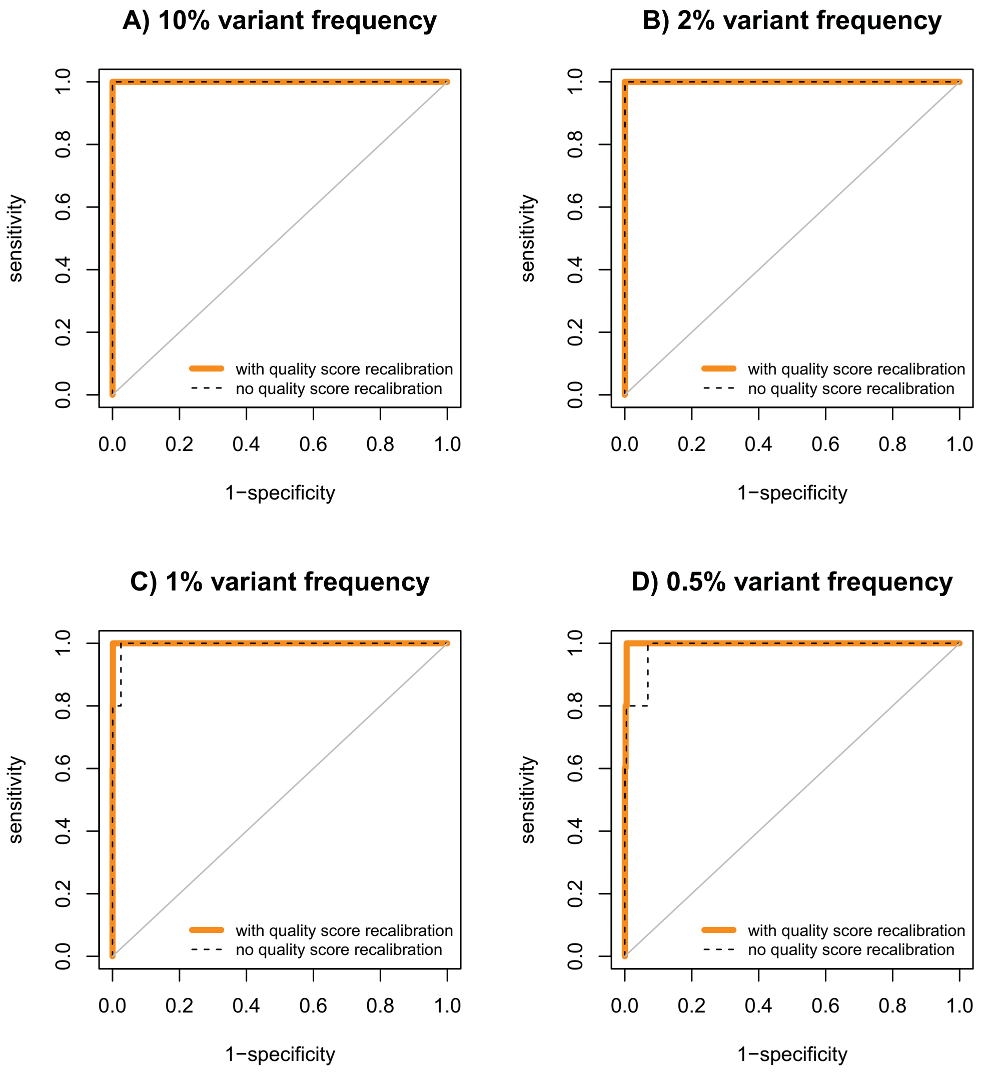

ROC curves for QQ−SNV without variant frequency filtering on HCV plasmid mixture datasets (all paired−end reads) with different spiked−in variant frequencies A) 10% B) 2% C) 1% and D) 0.5%, and comparing quality score recalibration *vs.* no quality score recalibration.

Figure S11. QQnorm.dir

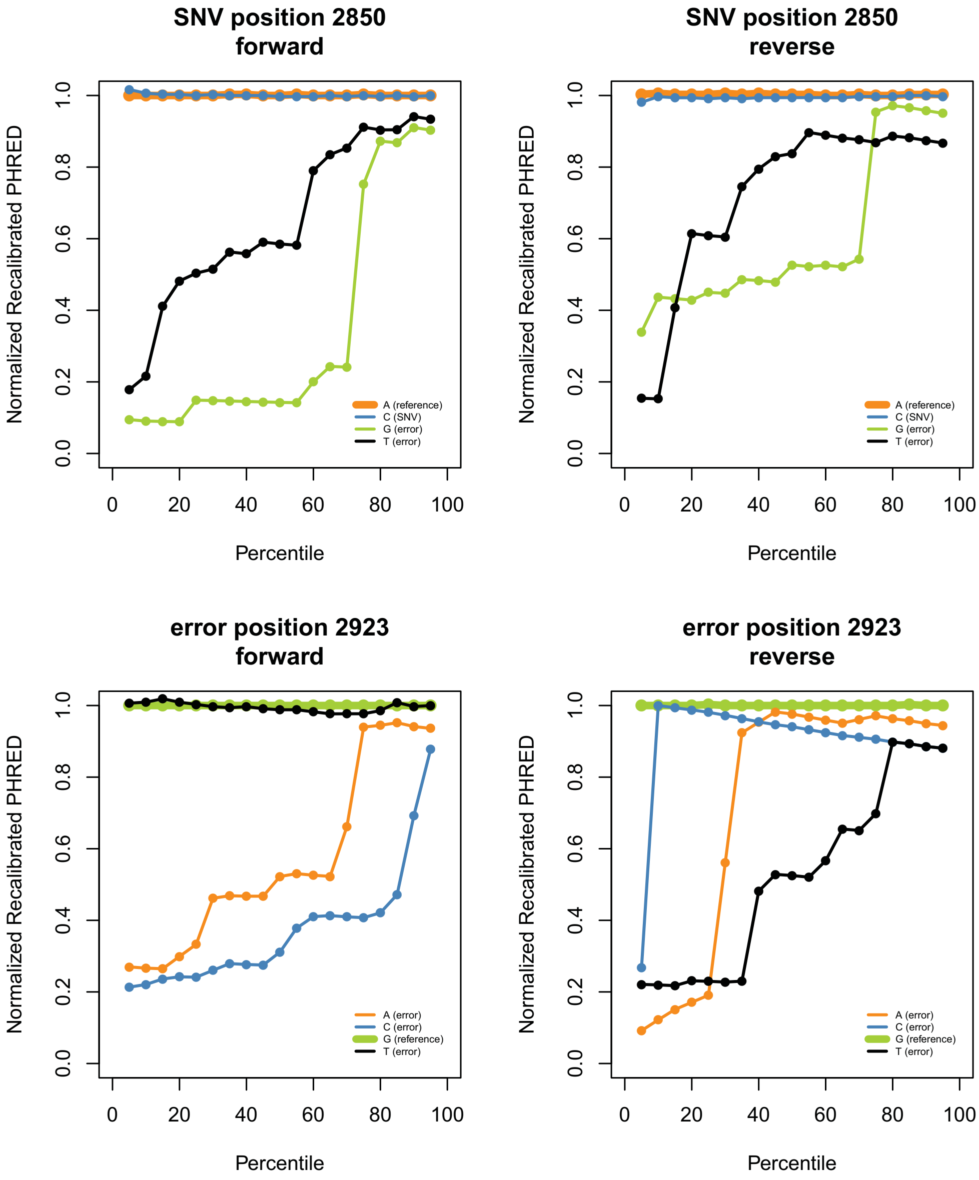

Normalized recalibrated PHRED scores per nucleotide (A/C/G/T) in the forward and reverse read mapping and calculated for nineteen percentiles (5th,10th,...,95th) at SNV position 2850 and at error position 2923 for training sample 1 (out of 96 *in silico* read sets).

Figure S12. QQ.dir

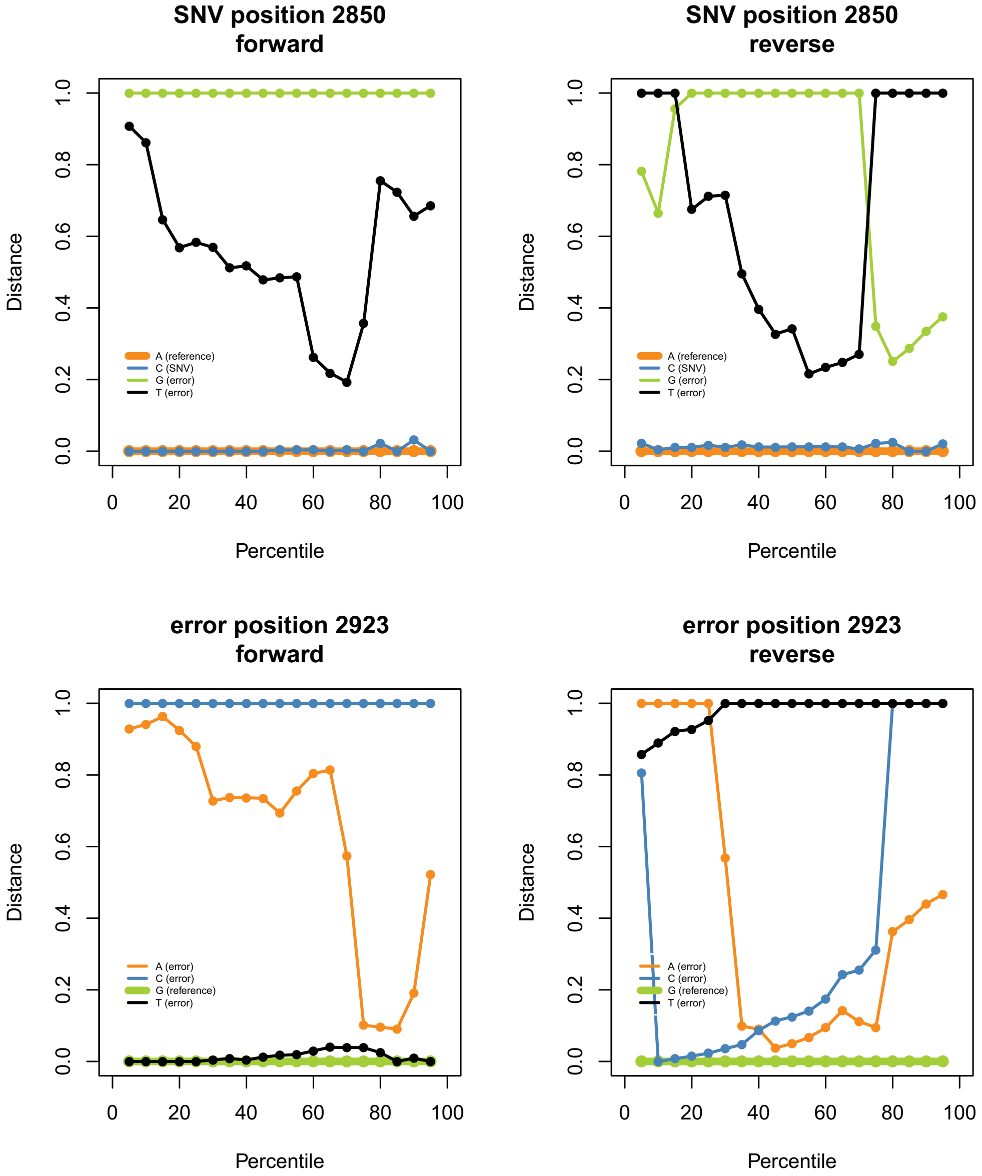

Distance per nucleotide (A/C/G/T) in the range [0,1] in the forward and reverse read mapping and calculated for nineteen percentiles (5th,10th,...,95th) relative to the nucleotide with worst quality at SNV position 2850 and at error position 2923 for training sample 1 (out of 96 *in silico* read sets).

Figure S13. D.dir

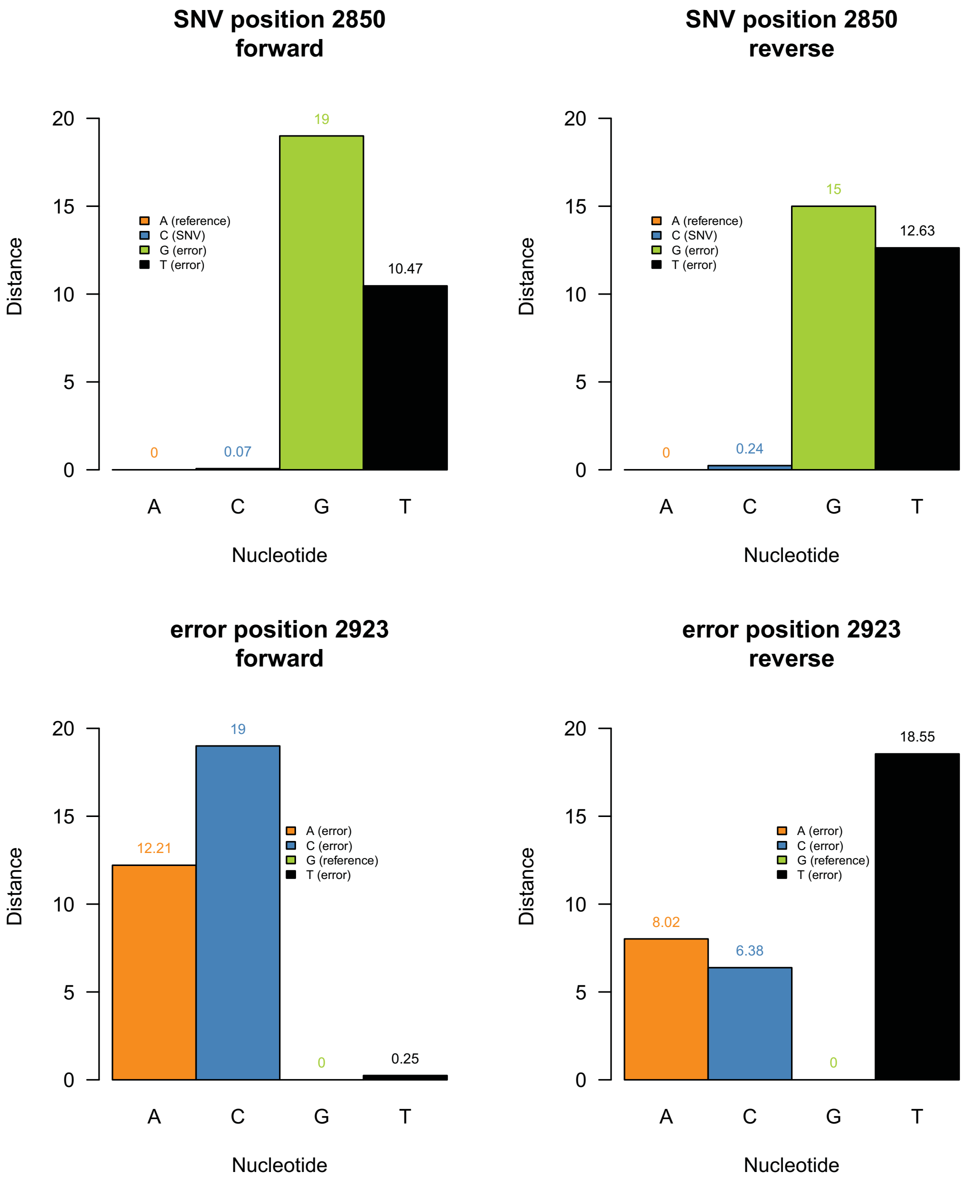

Distance per nucleotide (A/C/G/T) in the range [0,19] in the forward and reverse read mapping and calculated as the sum of distances for nineteen percentiles (5th,10th,...,95th) relative to the nucleotide with worst quality at SNV position 2850 and at error position 2923 for training sample 1 (out of 96 *in silico* read sets).

Figure S14.
Training of the QQ−SNV model by weighted logistic regression

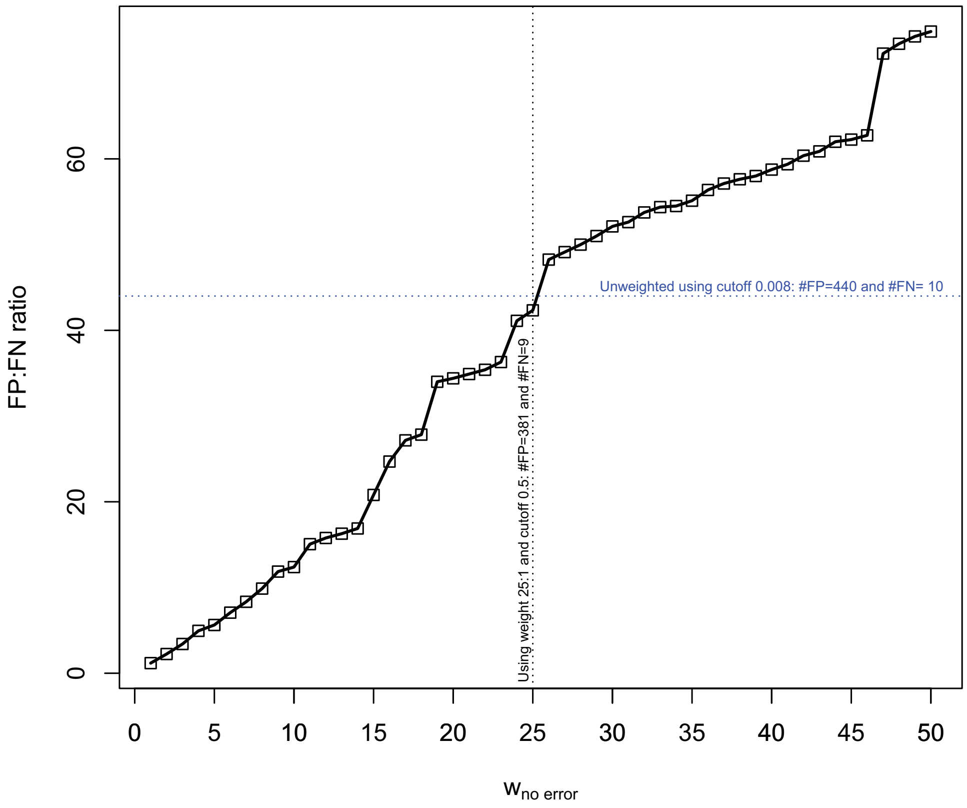

False positive : false negative ratio on the training data when varying the logistic regression weight $w_{no\ error}$ (w_{error}=1) and using cutoff 0.5 for error/no error classification (QQ−SNV_D).

Figure S15.
Performance on plasmid mixture test datasets

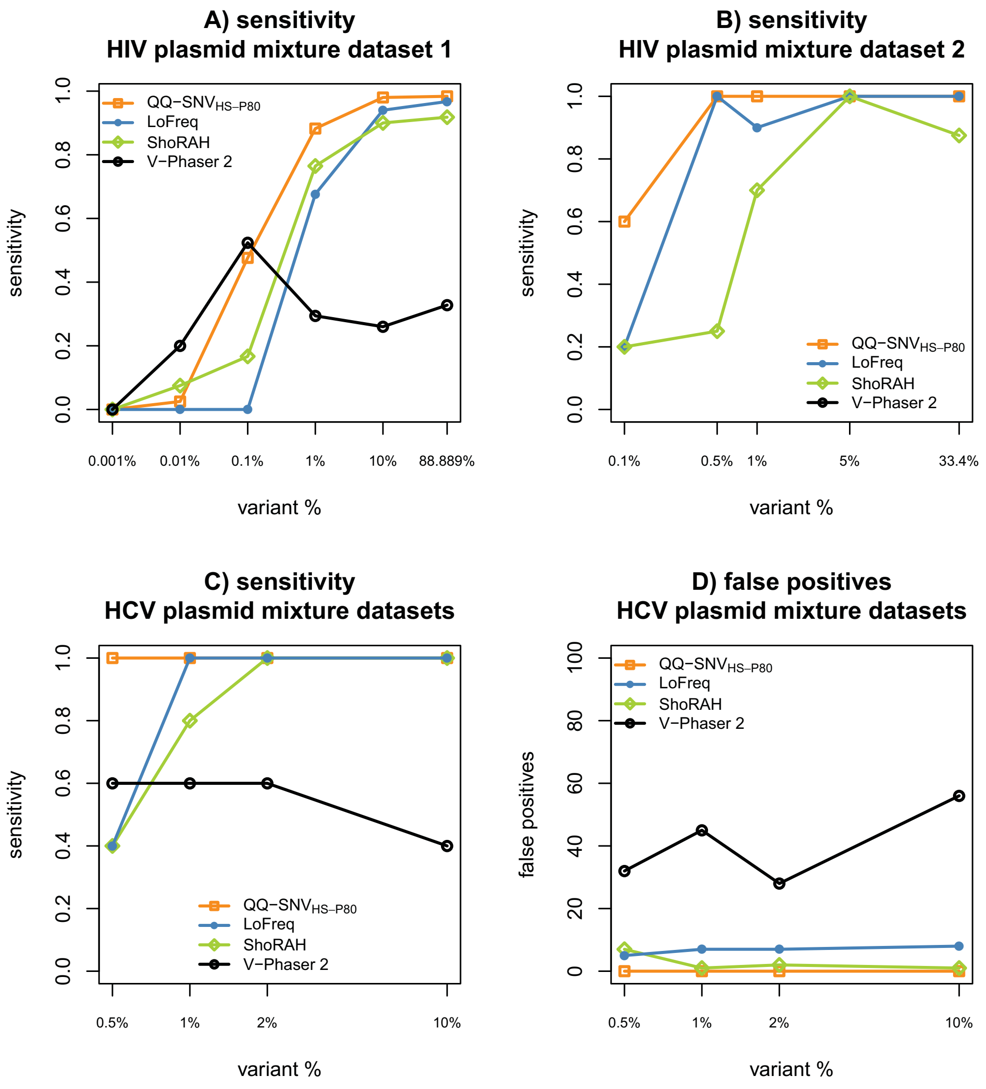

Performance of QQ−SNV$_{HS-P80}$, LoFreq, ShoRAH, and V−Phaser 2 on plasmid mixture test datasets by variant frequency (curves for Table 3−5). A) sensitivity on HIV plasmid mixture dataset 1 (Table 3). B) sensitivity on HIV plasmid mixture dataset 2 (Table 4) (variant frequencies shown for n>5 only). C) and D) sensitivity and false positives on HCV plasmid mixture datasets (all paired−end reads) (Table 5).

Figure S16.
ROC curves for QQ−SNV without variant frequency filtering
at different coverage depths

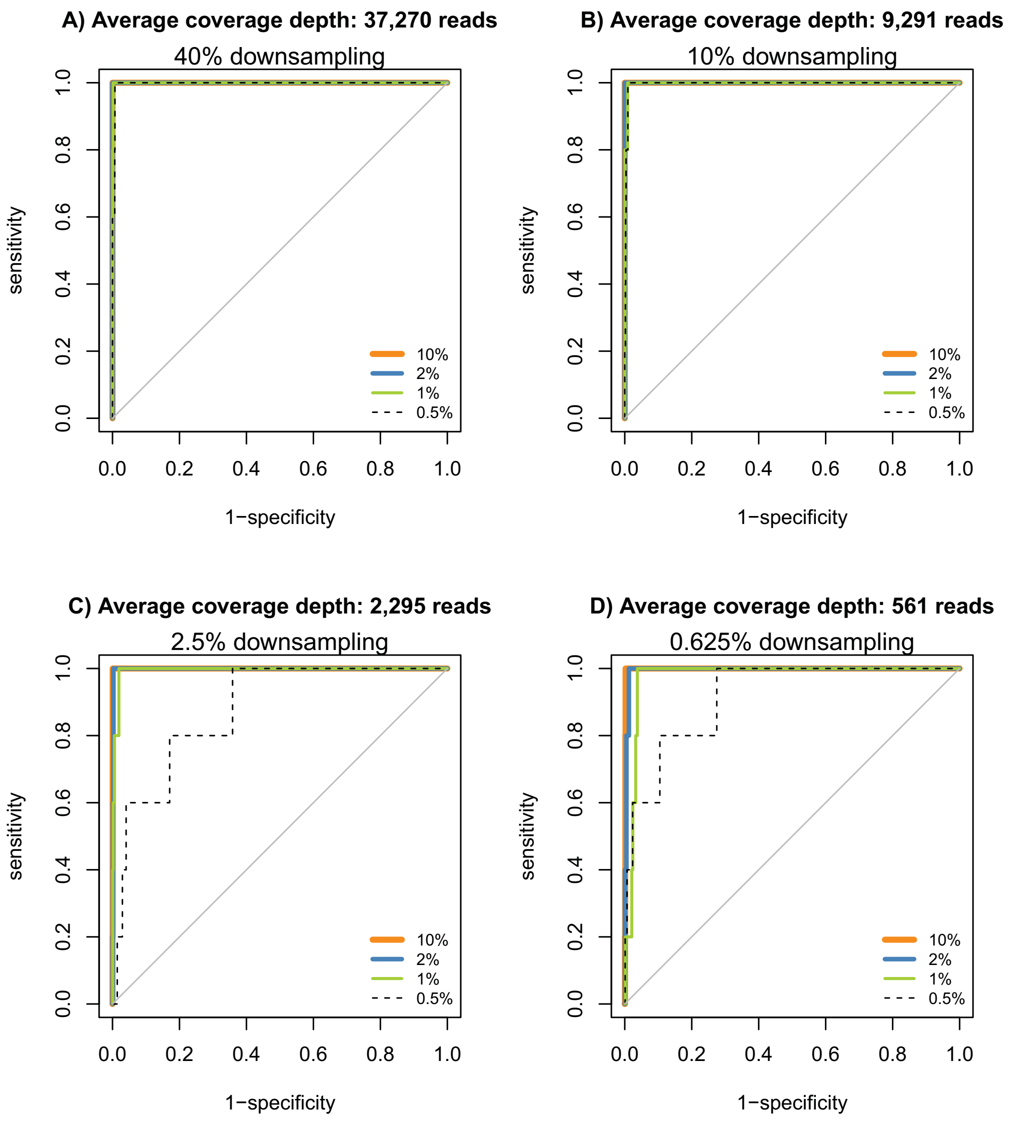

ROC curves for QQ−SNV without variant frequency filtering on HCV plasmid mixture datasets (all paired−end reads) at different coverage depths by downsampling A) 40% B) 10% C) 2.5% and D) 0.625%.

Figure S17.
ROC curves for QQ−SNV with P50 variant frequency filtering at different coverage depths

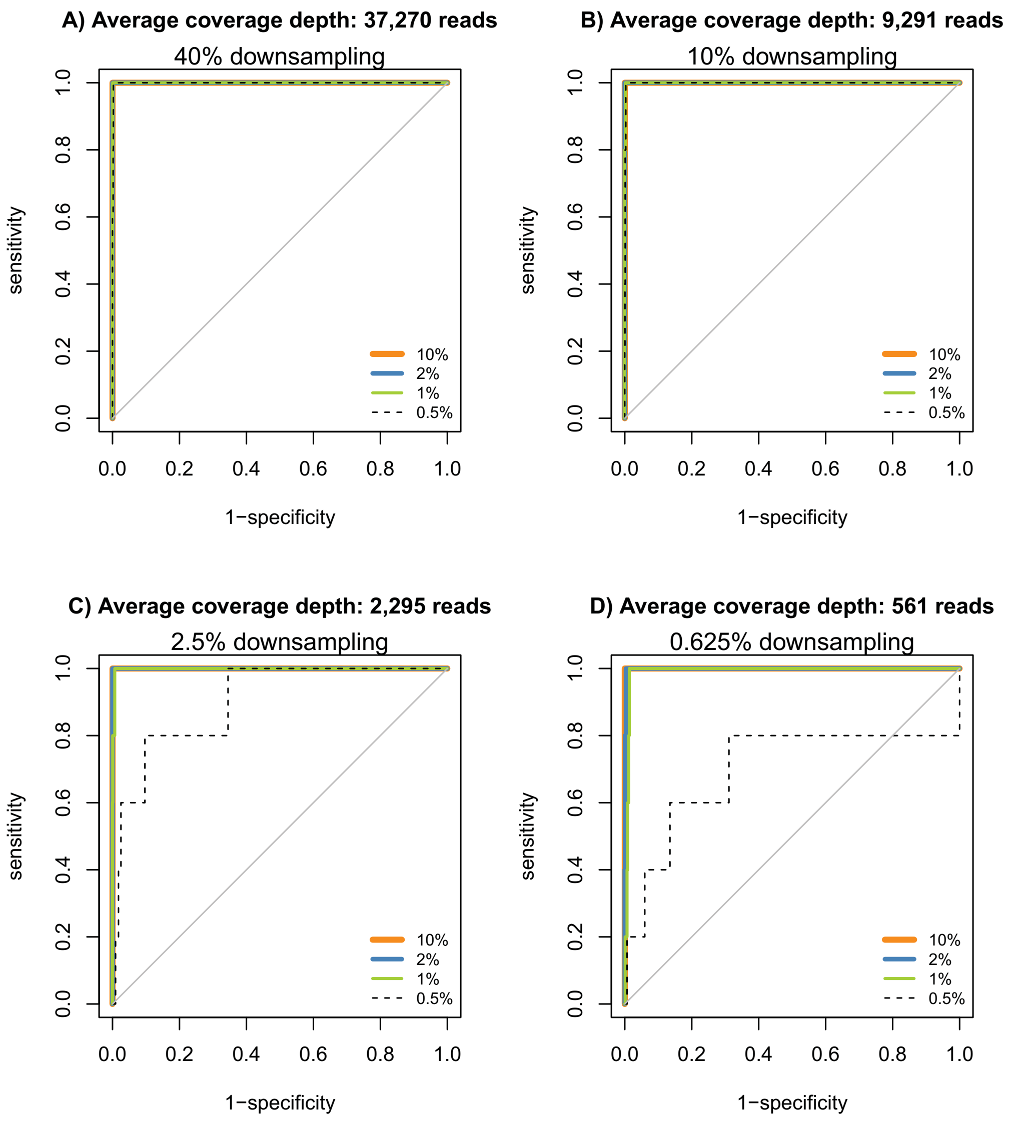

ROC curves for QQ−SNV with P50 variant frequency filtering on HCV plasmid mixture datasets (all paired−end reads) at different coverage depths by downsampling A) 40% B) 10% C) 2.5% and D) 0.625%.

Figure S18.
ROC curves for QQ−SNV with P75 variant frequency filtering at different coverage depths

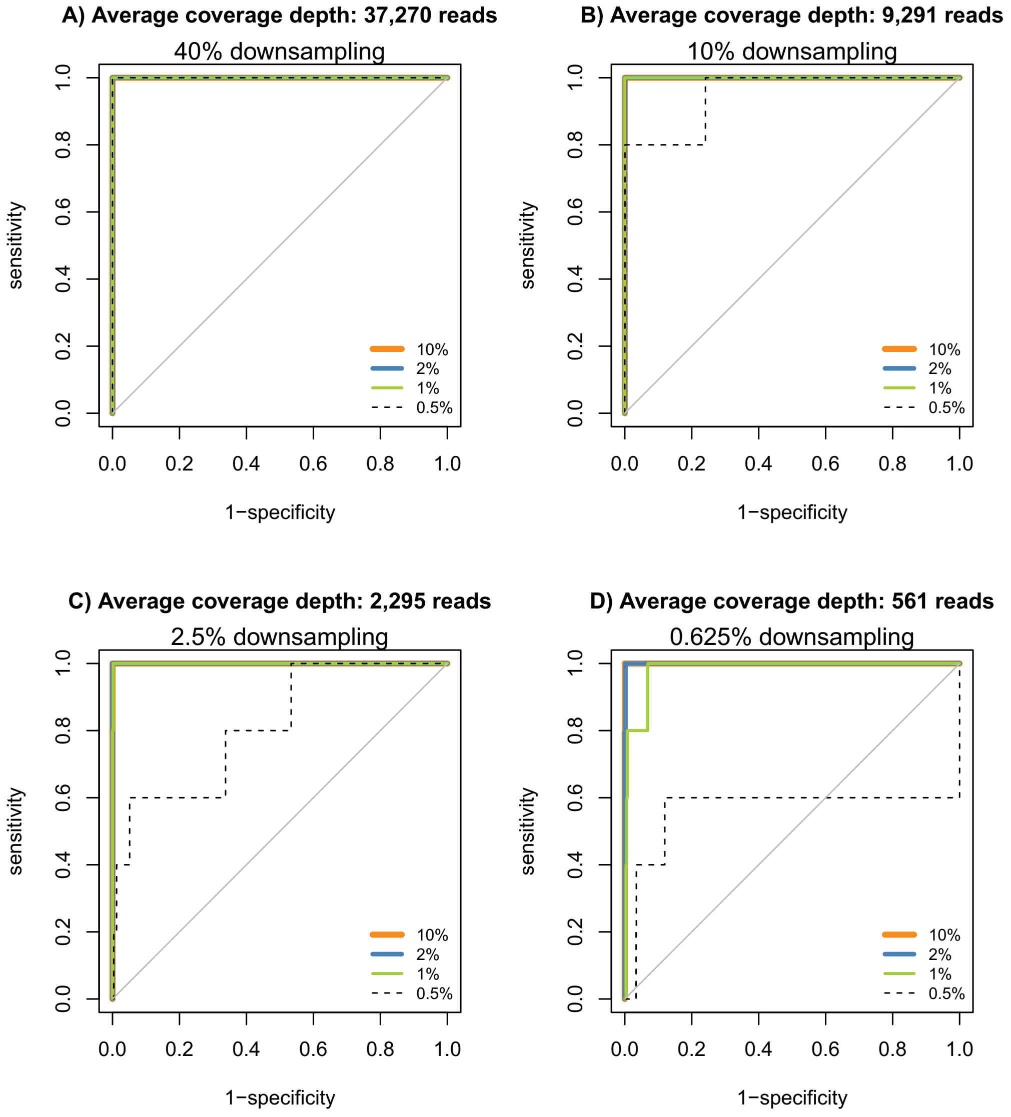

ROC curves for QQ−SNV with P75 variant frequency filtering on HCV plasmid mixture datasets (all paired−end reads) at different coverage depths by downsampling A) 40% B) 10% C) 2.5% and D) 0.625%.

Figure S19.
ROC curves for QQ−SNV with P80 variant frequency filtering
at different coverage depths

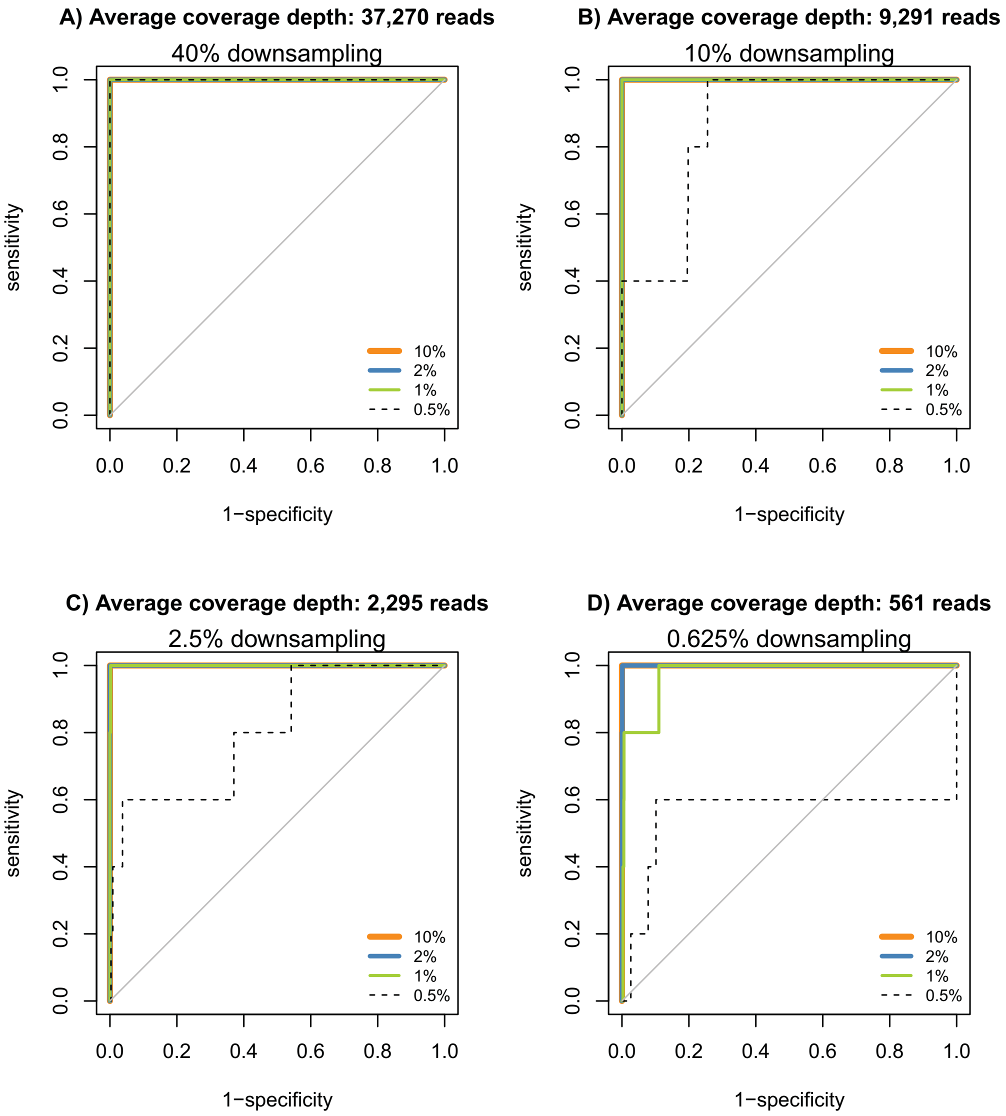

ROC curves for QQ−SNV with P80 variant frequency filtering on HCV plasmid mixture datasets (all paired−end reads) at different coverage depths by downsampling A) 40% B) 10% C) 2.5% and D) 0.625%.
