# Supplementary material for: QQ-SNV: single nucleotide variant detection at low frequency by comparing the quality quantiles
Source: BMC Bioinformatics. 2015 Nov 10;16:379. doi: 10.1186/s12859-015-0812-9 (PMC4641353; doi:10.1186/s12859-015-0812-9)
Supplement: Additional file 2: — Supplementary Tables. Sequence and error profile of HIV plasmids (Tables S1-S3). Training performance of QQ-SNV (Table S4), performance of QQ-SNV on in silico HIV plasmid mixture ground set (Table S5) and performance of QQ-SNV on HIV, HCV and H1N1 test sets (Tables S6-S10). (PDF 84 kb) [file 12859_2015_812_MOESM2_ESM.pdf]

Table S1. Presence of SNVs in the HIV-1 PR-RT region of five plasmids (nucleotide position 2253-3749)

Sample	Nucleotide position											
	2259	2850	2852	2857	2858	2927	3048	3090	3091	3675	3677	3693
HXB2	G	A	A	A	A	G	A	T	A	A	C	A
plasmid1	A	.	.	.	.	A	G	.	.	.	.	.
plasmid2	A	C	C	G	.	A	.	.	G	.	.	.
plasmid3	A	.	.	.	T	A	.	G	G	.	.	.
plasmid4	A	.	.	.	.	A	.	.	.	T	A	.
plasmid5	A	.	.	.	.	A	.	.	.	.	.	G

Table S2. Number of reads in the FASTQ/1 GAIIx training data

Sample	#reads forward	#reads reverse	Total
plasmid1	154,924	145,967	300,891
plasmid2	56,891	57,653	114,544
plasmid3	131,701	124,689	256,390
plasmid4	129,642	128,343	257,985
plasmid5	127,059	126,293	253,352
Total	600,217	582,945	1,183,162

Table S3. Number of nucleotides in the FASTQ/1 GAIIx training data and PHRED quality score per plasmid

Sample	Error[a]	No Error[b]	Total[c]	PHRED ($-10 \mathrm{Log}_{10}(\mathrm{P(error)})$)[d]
plasmid1	30,666	14,747,350	14,778,016	26.83
plasmid2	23,780	5,647,434	5,671,214	23.77
plasmid3	79,993	12,630,905	12,710,898	22.01
plasmid4	64,048	12,677,225	12,741,273	22.99
plasmid5	67,526	12,475,449	12,542,975	22.69
Total	266,013	58,178,363	58,444,376	23.42

[a]total number of nucleotide errors in the PR-RT region 2253-3749 per plasmid.

[b]total number of nucleotides without error in the PR-RT region 2253-3749 per plasmid.

[c]total number of nucleotides in the PR-RT region 2253-3749 per plasmid.

[d]PHRED quality score per plasmid.

Table S4. Training performance of QQ-SNV on HIV-1 *in silico* plasmid mixture data (96 samples)

	QQ-SNV (default)	
	with quality score recalibration	no quality score recalibration
Variant %	sensitivity[a]	sensitivity[a]
100.0%	100.0% (192/192)	100.0% (192/192)
93.0%	100.0% (6/6)	100.0% (6/6)
68.0%	100.0% (6/6)	100.0% (6/6)
64.5%	100.0% (6/6)	100/0% (6/6)
63.5%	100.0% (6/6)	100.0% (6/6)
63.0%	100.0% (144/144)	100.0% (144/144)
35.0%	100.0% (12/12)	100.0% (12/12)
31.5%	100.0% (12/12)	100.0% (12/12)
30.5%	100.0% (12/12)	100.0% (12/12)
30.0%	100.0% (180/180)	100.0% (180/180)
6.5%	100.0% (12/12)	100.0% (12/12)
5.5%	100.0% (12/12)	100.0% (12/12)
5.0%	100.0% (180/180)	100.0% (180/180)
2.0%	100.0% (12/12)	100.0% (12/12)
1.5%	100.0% (180/180)	91.1% (164/180)
0.5%	95.0% (171/180)	51.1% (92/180)
all	99.2% (1143/1152)	91.0% (1048/1152)

QQ-SNV (default)	sensitivity[a]	specificity[b]	PPV[c]	FP[d]
with quality score recalibration	99.2%	99.9%	75.0%	381
no quality score recalibration	91.0%	99.4%	29.4%	2517

[a]sensitivity is the percentage of *true* variants that were correctly called as SNV.

[b]specificity is the percentage of variants correctly identified as errors.

[c]PPV is the percentage of variants called as SNV that were *true*.

[d]FP is the total number of variants incorrectly called as SNV in any of the 96 training samples.

Table S5. Performance of QQ-SNV on HIV-1 *in silico* plasmid mixture data (ground set of 960 samples)

	$QQ\text{-}SNV_D$	$QQ\text{-}SNV_{HS}$	$QQ\text{-}SNV_{HS\text{-}P50}$[a]	$QQ\text{-}SNV_{HS\text{-}P75}$[a]	$QQ\text{-}SNV_{HS\text{-}P90}$[a]
Variant %	sens.[b]	sens.[b]	sens.[b]	sens.[b]	sens.[b]
<0.5%	96.3%	100%	100%	100%	99.7%
0.5% - <1%	98.0%	100%	100%	100%	100%
1% - <1.5%	100%	100%	100%	100%	100%
1.5% - <2%	100%	100%	100%	100%	100%
2% - <5%	100%	100%	100%	100%	100%
5% - <10%	100%	100%	100%	100%	100%
10% - <50%	100%	100%	100%	100%	100%
≥50%	100%	100%	100%	100%	100%
	spec.[c]	spec.[c]	spec.[c]	spec.[c]	spec.[c]
	98.9%	85.6%	88.3%	90.6%	93.9%
	PPV[d]	PPV[d]	PPV[d]	PPV[d]	PPV[d]
	19.6%	1.82%	2.24%	2.77%	4.19%
	FP[e]	FP[e]	FP[e]	FP[e]	FP[e]
	46954	621146	503284	404489	263047

[a]50th/75th/90th percentile of the $QQ\text{-}SNV_{HS}$ "error" distribution was used as frequency cutoff to decrease the number of false positives obtained with $QQ\text{-}SNV_{HS}$.
[b]sens. is the percentage of *true* variants that were correctly called as SNV.
[c]spec. is the percentage of variants correctly identified as errors.
[d]PPV is the percentage of variants called as SNV that were *true*.
[e]FP is the total number of variants incorrectly called as SNV in any of the 960 samples.

Table S6. Performance of QQ-SNV on HIV plasmid mixture dataset 1

	$QQ\text{-}SNV_D$	$QQ\text{-}SNV_{HS}$	$QQ\text{-}SNV_{HS\text{-}P50}$[a]	$QQ\text{-}SNV_{HS\text{-}P75}$[a]	$QQ\text{-}SNV_{HS\text{-}P80}$[a]	$QQ\text{-}SNV_{HS\text{-}P85}$[a]	$QQ\text{-}SNV_{HS\text{-}P90}$[a]	$QQ\text{-}SNV_{HS\text{-}P95}$[a]
Variant %	sens.[b]	sens.[b]	sens.[b]	sens.[b]	sens.[b]	sens.[b]	sens.[b]	sens.[b]
88.889%	57/61	60/61	60/61	60/61	60/61	60/61	60/61	60/61
10%	44/50	49/50	49/50	49/50	49/50	49/50	49/50	48/50
1%	28/34	30/34	30/34	30/34	30/34	30/34	30/34	29/34
0.1%	2/42	23/42	23/42	23/42	20/42	18/42	10/42	3/42
0.01%	0/40	6/40	4/40	1/40	1/40	0/40	0/40	0/40
0.001%	0/30	0/30	0/30	0/30	0/30	0/30	0/30	0/30
	FP[c]	FP[c]	FP[c]	FP[c]	FP[c]	FP[c]	FP[c]	FP[c]
	2	51	21	7	4	1	1	1
	time[de]	time[de]	time[de]	time[de]	time[de]	time[de]	time[de]	time[de]
	14m11s	14m11s	14m11s	14m11s	14m11s	14m11s	14m11s	14m11s

[a]$50^{th}/75^{th}/80^{th}/85^{th}/90^{th}/95^{th}$ percentile of the $QQ\text{-}SNV_{HS}$ distribution of "error" frequencies was used as frequency cutoff to decrease the number of false positives obtained with $QQ\text{-}SNV_{HS}$.
[b]sens. is the percentage of *true* variants that were correctly called as SNV.
[c]FP is the number of variants incorrectly called as SNV.
[d]computation time in minutes (m) and seconds (s).
[e]Windows 7 64bit, 8GB RAM, 3.2GHz

Table S7. Performance of QQ-SNV on HIV plasmid mixture dataset 2

	$QQ\text{-}SNV_{D}$	$QQ\text{-}SNV_{HS}$	$QQ\text{-}SNV_{HS\text{-}P50}$[a]	$QQ\text{-}SNV_{HS\text{-}P75}$[a]	$QQ\text{-}SNV_{HS\text{-}P80}$[a]	$QQ\text{-}SNV_{HS\text{-}P85}$[a]	$QQ\text{-}SNV_{HS\text{-}P90}$[a]	$QQ\text{-}SNV_{HS\text{-}P95}$[a]
Variant %	sens.[b]	sens.[b]	sens.[b]	sens.[b]	sens.[b]	sens.[b]	sens.[b]	sens.[b]
40.0%	2/2	2/2	2/2	2/2	2/2	2/2	2/2	2/2
39.9%	1/1	1/1	1/1	1/1	1/1	1/1	1/1	1/1
39.5%	1/1	1/1	1/1	1/1	1/1	1/1	1/1	1/1
38.9%	1/1	1/1	1/1	1/1	1//1	1/1	1/1	1/1
38.4%	2/2	2/2	2/2	2/2	2/2	2/2	2/2	2/2
34.4%	2/2	2/2	2/2	2/2	2/2	2/2	2/2	2/2
34.0%	1/1	1/1	1/1	1/1	1/1	1/1	1/1	1/1
33.5%	1/1	1/1	1/1	1/1	1/1	1/1	1/1	1/1
33.4%	8/8	8/8	8/8	8/8	8/8	8/8	8/8	8/8
6.6%	1/1	1/1	1/1	1/1	1/1	1/1	1/1	1/1
6.1%	1/1	1/1	1/1	1/1	1/1	1/1	1/1	1/1
5.1%	1/1	1/1	1/1	1/1	1/1	1/1	1/1	1/1
5.0%	8/8	8/8	8/8	8/8	8/8	8/8	8/8	8/8
1.0%	10/10	10/10	10/10	10/10	10/10	10/10	10/10	10/10
0.6%	0/1	1/1	1/1	1/1	1/1	1/1	1/1	1/1
0.5%	5/8	8/8	8/8	8/8	8/8	8/8	8/8	8/8
0.1%	1/10	6/10	6/10	6/10	6/10	6/10	6/10	6/10
	FP[c]	FP[c]	FP[c]	FP[c]	FP[c]	FP[c]	FP[c]	FP[c]
	0	1	1	1	1	1	1	1
	time[de]	time[de]	time[de]	time[de]	time[de]	time[de]	time[de]	time[de]
	2h28m43s	2h28m43s	2h28m43s	2h28m43s	2h28m43s	2h28m43s	2h28m43s	2h28m43s

[a] $50^{th}/75^{th}/80^{th}/85^{th}/90^{th}/95^{th}$ percentile of the $QQ\text{-}SNV_{HS}$ distribution of "error" frequencies was used as frequency cutoff to decrease the number of false positives obtained with $QQ\text{-}SNV_{HS}$.
[b] sens. is the percentage of *true* variants that were correctly called as SNV.
[c] FP is the number of variants incorrectly called as SNV.
[d] computation time in hours (h), minutes (m) and seconds (s).
[e] Windows 7 64bit, 8GB RAM, 3.2GHz.

Table S8. Performance of QQ-SNV on HCV plasmid mixture datasets

		$QQ\text{-}SNV_{D}$			$QQ\text{-}SNV_{HS}$			$QQ\text{-}SNV_{HS\text{-}P50}$ [a]			$QQ\text{-}SNV_{HS\text{-}P75}$ [a]		
Variant %	Pair	sens. [b]	FP [c]	time [gh]	sens. [b]	FP [c]	time [gh]	sens. [b]	FP [c]	time [gh]	sens. [b]	FP [c]	time [gh]
0.50%	1 [d]	5/5	10	2m27s	5/5	112	2m27s	5/5	68	2m27s	5/5	46	2m27s
0.50%	2 [e]	0/5	0	2m18s	4/5	3	2m18s	4/5	0	2m18s	0/5	0	2m18s
0.50%	1+2 [f]	1/5	0	4m42s	5/5	12	4m42s	5/5	4	4m42s	5/5	0	4m42s
1%	1 [d]	5/5	11	2m56s	5/5	122	2m56s	5/5	78	2m56s	5/5	59	2m56s
1%	2 [e]	1/5	0	3m1s	5/5	5	3m1s	5/5	0	3m1s	5/5	0	3m1s
1%	1+2 [f]	3/5	1	5m48s	5/5	14	5m48s	5/5	2	5m48s	5/5	0	5m48s
2%	1 [d]	5/5	13	2m35s	5/5	146	2m35s	5/5	100	2m35s	5/5	70	2m35s
2%	2 [e]	3/5	0	2m32s	5/5	5	2m32s	5/5	0	2m32s	5/5	0	2m32s
2%	1+2 [f]	5/5	0	4m59s	5/5	14	4m59s	5/5	3	4m59s	5/5	0	4m59s
10%	1 [d]	5/5	6	3m5s	5/5	112	3m5s	5/5	71	3m5s	5/5	49	3m5s
10%	2 [e]	5/5	0	2m30s	5/5	5	2m30s	5/5	1	2m30s	5/5	0	2m30s
10%	1+2 [f]	5/5	0	5m29s	5/5	7	5m29s	5/5	2	5m29s	5/5	0	5m29s

		$QQ\text{-}SNV_{HS\text{-}P80}$ [a]			$QQ\text{-}SNV_{HS\text{-}P85}$ [a]			$QQ\text{-}SNV_{HS\text{-}P90}$ [a]			$QQ\text{-}SNV_{HS\text{-}P95}$ [a]		
Variant %	Pair	sens. [b]	FP [c]	time [gh]	sens. [b]	FP [c]	time [gh]	sens. [b]	FP [c]	time [gh]	sens. [b]	FP [c]	time [gh]
0.50%	1 [d]	5/5	38	2m27s	5/5	29	2m27s	5/5	17	2m27s	5/5	8	2m27s
0.50%	2 [e]	0/5	0	2m18s	0/5	0	2m18s	0/5	0	2m18s	0/5	0	2m18s
0.50%	1+2 [f]	5/5	0	4m42s	3/5	0	4m42s	1/5	0	4m42s	1/5	0	4m42s
1%	1 [d]	5/5	53	2m56s	5/5	47	2m56s	5/5	33	2m56s	5/5	10	2m56s
1%	2 [e]	5/5	0	3m1s	5/5	0	3m1s	3/5	0	3m1s	1/5	0	3m1s
1%	1+2 [f]	5/5	0	5m48s	5/5	0	5m48s	5/5	0	5m48s	5/5	0	5m48s
2%	1 [d]	5/5	64	2m35s	5/5	55	2m35s	5/5	34	2m35s	5/5	15	2m35s
2%	2 [e]	5/5	0	2m32s	5/5	0	2m32s	5/5	0	2m32s	5/5	0	2m32s
2%	1+2 [f]	5/5	0	4m59s	5/5	0	4m59s	5/5	0	4m59s	5/5	0	4m59s
10%	1 [d]	5/5	42	3m5s	5/5	29	3m5s	5/5	18	3m5s	5/5	9	3m5s
10%	2 [e]	5/5	0	2m30s	5/5	0	2m30s	5/5	0	2m30s	5/5	0	2m30s
10%	1+2 [f]	5/5	0	5m29s	5/5	0	5m29s	5/5	0	5m29s	5/5	0	5m29s

[a] $50^{th}/75^{th}/80^{th}/85^{th}/90^{th}/95^{th}$ percentile of the $QQ\text{-}SNV_{HS}$ distribution of “error” frequencies was used as frequency cutoff to decrease the number of false positives obtained with $QQ\text{-}SNV_{HS}$.

[b] sens. is the percentage of *true* variants that were correctly called as SNV.

[c] FP is the number of variants incorrectly called as SNV.

[d] Reads 1 of paired-end reads.

[e] Reads 2 of paired-end reads (sequenced after paired-end turn).

[f] All paired-end reads.

[g] computation time in minutes (m) and seconds (s).

[h] Windows 7 64 bit, 8GB RAM, 3.2GHz.

Table S9. Performance at different coverage depths on HCV plasmid mixture datasets (all paired-end reads)

Downsampling	Variant %	Average coverage depth (number of reads)	QQ-SNV$_D$ sens.[b]	QQ-SNV$_D$ FP[c]	QQ-SNV$_{HS}$ sens.[b]	QQ-SNV$_{HS}$ FP[c]	QQ-SNV$_{HS-P50}$[a] sens.[b]	QQ-SNV$_{HS-P50}$[a] FP[c]	QQ-SNV$_{HS-P75}$[a] sens.[b]	QQ-SNV$_{HS-P75}$[a] FP[c]
40%	0.50%	32,641	1/5	0	5/5	15	5/5	5	5/5	0
40%	1%	41,691	3/5	1	5/5	14	5/5	2	5/5	0
40%	2%	35,202	5/5	0	5/5	16	5/5	3	5/5	0
40%	10%	39,544	5/5	0	5/5	9	5/5	2	5/5	0
10%	0.50%	8,142	0/5	0	5/5	30	5/5	9	4/5	1
10%	1%	10,391	4/5	1	5/5	24	5/5	4	5/5	0
10%	2%	8,777	5/5	1	5/5	35	5/5	8	5/5	3
10%	10%	9,856	5/5	0	5/5	19	5/5	5	5/5	1
2.5%	0.50%	2,012	0/5	5	1/5	34	1/5	19	1/5	7
2.5%	1%	2,568	3/5	4	5/5	31	5/5	11	5/5	3
2.5%	2%	2,167	5/5	5	5/5	34	5/5	14	5/5	6
2.5%	10%	2,434	5/5	2	5/5	23	5/5	7	5/5	5
0.625%	0.50%	490	1/5	9	2/5	21	0/5	2	0/5	1
0.625%	1%	631	1/5	21	3/5	39	3/5	14	2/5	6
0.625%	2%	527	4/5	12	5/5	23	5/5	7	5/5	3
0.625%	10%	595	5/5	7	5/5	22	5/5	4	5/5	3

Downsampling	Variant %	Average coverage depth (number of reads)	QQ-SNV$_{HS-P80}$[a] sens.[b]	QQ-SNV$_{HS-P80}$[a] FP[c]	LoFreq sens.[b]	LoFreq FP[c]	ShoRAH sens.[b]	ShoRAH FP[c]	V-Phaser 2 sens.[b]	V-Phaser 2 FP[c]
40%	0.50%	32,641	5/5	0	1/5	2	4/5	0	4/5	0
40%	1%	41,691	5/5	0	5/5	3	5/5	0	3/5	2
40%	2%	35,202	5/5	0	5/5	3	5/5	1	4/5	2
40%	10%	39,544	5/5	0	5/5	7	5/5	1	2/5	4
10%	0.50%	8,142	2/5	0	0/5	0	5/5	0	3/5	0
10%	1%	10,391	5/5	0	5/5	2	5/5	0	3/5	0
10%	2%	8,777	5/5	3	5/5	0	5/5	0	4/5	0
10%	10%	9,856	5/5	1	5/5	2	5/5	1	5/5	0
2.5%	0.50%	2,012	1/5	5	0/5	0	0/5	0	2/5	0
2.5%	1%	2,568	5/5	3	2/5	0	5/5	0	5/5	0
2.5%	2%	2,167	5/5	6	5/5	0	5/5	0	5/5	0
2.5%	10%	2,434	5/5	3	5/5	1	5/5	0	5/5	0
0.625%	0.50%	490	0/5	1	0/5	0	0/5	0	0/5	0
0.625%	1%	631	2/5	5	0/5	0	0/5	0	2/5	0
0.625%	2%	527	5/5	2	3/5	0	5/5	0	5/5	0
0.625%	10%	595	5/5	2	5/5	0	5/5	0	5/5	0

[a]$50^{th}/75^{th}/80^{th}$ percentile of the QQ-SNV$_{HS}$ distribution of “error” frequencies was used as frequency cutoff to decrease the number of false positives obtained with QQ-SNV$_{HS}$.

[b]sens. is the percentage of *true* variants that were correctly called as SNV.

[c]FP is the number of variants incorrectly called as SNV.

Table S10. Performance of QQ-SNV on H1N1 clinical sample

		QQ-SNV$_{D}$			QQ-SNV$_{HS}$			QQ-SNV$_{HS\text{-}P50}$ [a]			QQ-SNV$_{HS\text{-}P75}$ [a]		
	Pair	sens. [b]	FP [c]	time [gh]	sens. [b]	FP [c]	time [gh]	sens. [b]	FP [c]	time [gh]	sens. [b]	FP [c]	time [gh]
GAIIx	1 [d]	0/4	0	12m51s	4/4	2	12m51s	4/4	2	12m51s	4/4	2	12m51s
GAIIx	2 [e]	0/4	0	13m5s	4/4	1	13m5s	4/4	1	13m5s	4/4	1	13m5s
GAIIx	1+2 [f]	0/4	0	21m56s	4/4	4	21m56s	4/4	4	21m56s	4/4	4	21m56s
MiSeq	1 [d]	2/4	1	1m11s	4/4	8	1m11s	4/4	7	1m11s	4/4	5	1m11s
MiSeq	2 [e]	2/4	0	1m23s	4/4	2	1m23s	4/4	2	1m23s	4/4	2	1m23s
MiSeq	1+2 [f]	4/4	3	2m45s	4/4	8	2m45s	4/4	7	2m45s	4/4	6	2m45s

		QQ-SNV$_{HS\text{-}P80}$ [a]			QQ-SNV$_{HS\text{-}P85}$ [a]			QQ-SNV$_{HS\text{-}P90}$ [a]			QQ-SNV$_{HS\text{-}P95}$ [a]		
Illumina Sequencer	Pair	sens. [b]	FP [c]	time [gh]	sens. [b]	FP [c]	time [gh]	sens. [b]	FP [c]	time [gh]	sens. [b]	FP [c]	time [gh]
GAIIx	1 [d]	4/4	2	12m51s	4/4	2	12m51s	4/4	2	12m51s	4/4	1	12m51s
GAIIx	2 [e]	4/4	1	13m5s	4/4	1	13m5s	4/4	1	13m5s	4/4	0	13m5s
GAIIx	1+2 [f]	4/4	4	21m56s	4/4	4	21m56s	4/4	4	21m56s	4/4	4	21m56s
MiSeq	1 [d]	4/4	5	1m11s	4/4	4	1m11s	4/4	3	1m11s	4/4	2	1m11s
MiSeq	2 [e]	4/4	2	1m23s	4/4	2	1m23s	4/4	2	1m23s	4/4	0	1m23s
MiSeq	1+2 [f]	4/4	6	2m45s	4/4	5	2m45s	4/4	5	2m45s	4/4	1	2m45s

[a] 50th/75th/80th/85th/90th/95th percentile of the QQ-SNV$_{HS}$ distribution of "error" frequencies was used as frequency cutoff to decrease the number of false positives obtained with QQ-SNV$_{HS}$.

[b] sens. is the percentage of SNVs as identified in [12] that were called as SNV by QQ-SNV in region 405-425 of NA gene in H1N1 BN3 sample.

[c] FP is the number of "non-variants" in [12] that were called as SNV by QQ-SNV.

[d] Reads 1 of paired-end reads.

[e] Reads 2 of paired-end reads (sequenced after paired-end turn).

[f] All paired-end reads.

[g] computation time in minutes (m) and seconds (s).

[h] Windows 7 64 bit, 8GB RAM, 3.2GHz.
