# Supplementary material for: QQ-SNV: single nucleotide variant detection at low frequency by comparing the quality quantiles
Source: BMC Bioinformatics. 2015 Nov 10;16:379. doi: 10.1186/s12859-015-0812-9 (PMC4641353; doi:10.1186/s12859-015-0812-9)
Supplement: Additional file 3: — Supporting evidence for stability of coefficients in the QQ-SNV companion logistic regression recalibration model. (PDF 6 kb) [file 12859_2015_812_MOESM3_ESM.pdf]

Supporting evidence for stability of coefficients in quality score recalibration model

Bootstrap 95% confidence interval for model parameters of Table 1.

	**2.5 %**	**97.5 %**
Intercept	0.35955614	1.2820099
QUAL	-0.15842187	-0.1577792
RELPOS	-0.91237613	-0.8778055
