# Supplementary material for: QQ-SNV: single nucleotide variant detection at low frequency by comparing the quality quantiles
Source: BMC Bioinformatics. 2015 Nov 10;16:379. doi: 10.1186/s12859-015-0812-9 (PMC4641353; doi:10.1186/s12859-015-0812-9)

Supporting evidence for applicability of QQ-SNV on Illumina NGS data of any read length

Reference sequence: KC866600

Illumina Miseq 250bp
Analysis on all paired-end reads
True variants: 334C>T, 625A>T (data from [28])

QQ-SNV predicted SNV probability for the *true* variants for plasmid mix ratios 95:5, 99:1 and 99.9:0.1

	95:5	99:1	99.9:0.1	
Average coverage depth (number of reads)	26,663	23,659	29,090	
	P(SNV)	P(SNV	P(SNV)	
334C>T	0.9999761488	0.9997707282	0.5613726795	
625A>T	0.9999456703	0.9992583561	0.9989070416	625A>T filtered by frequency for QQ-$SNV_{HS\text{-}P80}$ at mix ratio 99.9:0.1

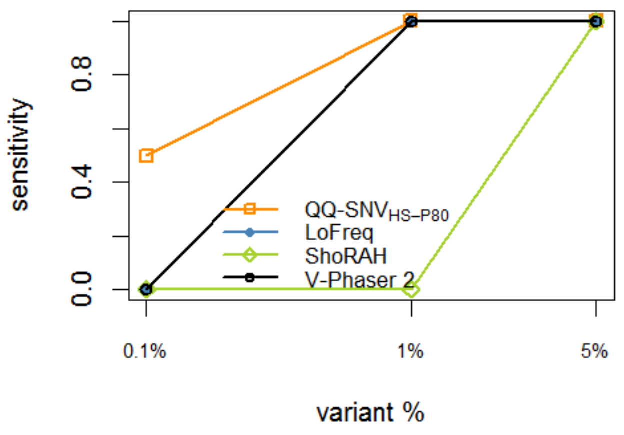

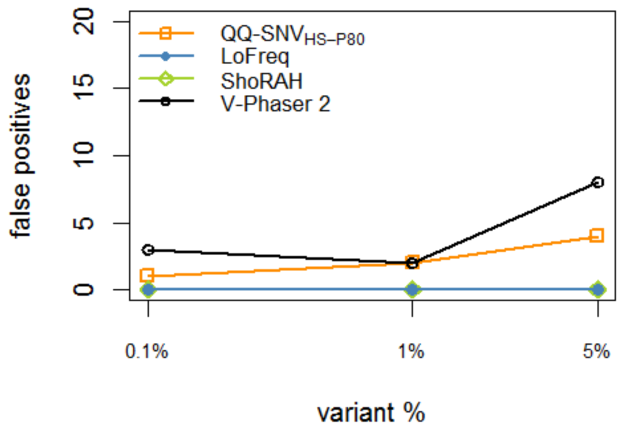

Supplement: Additional file 5: — Supporting evidence for applicability of QQ-SNV for SNV calling on Illumina NGS sequencing data of any read length. (PDF 32 kb) [file 12859_2015_812_MOESM5_ESM.pdf]
